# Phylogeography and genetic structure of *Papaver bracteatum* populations in Iran based on genotyping-by-sequencing (GBS)

**DOI:** 10.1038/s41598-024-67190-8

**Published:** 2024-07-15

**Authors:** Razieh Rahmati, Zahra Nemati, Mohammad Reza Naghavi, Simon Pfanzelt, Amir Rahimi, Ali Ghaderi Kanzagh, Frank R. Blattner

**Affiliations:** 1 Leibniz Institute of Plant Genetics and Crop Plant Research (IPK), Gatersleben, Germany; 2 Division of Biotechnology, Department of Agronomy and Plant Breeding, Agricultural and Natural Resources College, University of Tehran, Karaj, Iran; 3 Department of Plant Production and Genetics, Faculty of Agriculture and Natural Resources, Urmia University, Urmia, Iran; 4 Department of Biology, Science and Research Branch, Islamic Azad University, Tehran, Iran; 5 Present Address: Institute for Medical Microbiology and Hospital Hygiene, Goethe University Frankfurt,, Frankfurt/M., Germany; 6 Present Address: Bavarian Natural History Collections, Botanical Garden München-Nymphenburg, Munich, Germany

**Keywords:** Ecology, Evolution, Plant sciences

## Abstract

*Papaver bracteatum*, known for its high thebaine content and absence of morphine, has emerged as a promising alternative to opium poppy for codeine production. In this study, our objective was to create a diverse panel representing the natural variation of this species in Iran. To achieve this, we employed genotyping-by-sequencing to obtain genome-wide distributed single-nucleotide polymorphisms (SNPs) for phylogeographic analysis, population structure assessment, and evaluation of genetic diversity within *P. bracteatum* populations. A total of 244 *P. bracteatum* individuals from 13 distinct populations formed seven genetic groups, along with one highly admixed population. We observed a clear split between the populations inhabiting the Alborz Mts. in the east and Zagros Mts. in the west. In between these mountain ranges, the population of Kachal Mangan exhibited a high degree of genetic admixture between both genetic groups. At or after the end of the last glacial maximum, when climate conditions rapidly changed, all *P. bracteatum* populations experienced a strong demographic bottleneck reducing the already small effective population sizes further before they increased to their recent strengths. Our results suggest that the ongoing climate change together with human pressure on the species’ habitats and limited seed-dispersal ability are potential factors contributing today to rising genetic isolation of *P. bracteatum* populations. Our results provide genetic data that can be used for conservation measures to safeguard the species’ genetic diversity as a resource for future breeding approaches in this medicinally important species.

## Introduction

The genus *Papaver* L. has about 70–100 species, which are mainly found in temperate regions of the Northern Hemisphere^1,2^. It contains numerous annual and biennial species as well as a number of perennials. This genus is of commercial interest because of the medicinally important alkaloids present in several *Papaver* groups^3^. The economically most important alkaloids are morphine, codeine and thebaine. These alkaloids are known to occur in large quantity in the opium poppy, *Papaver somniferum* L^4–7^. Among these alkaloids, thebaine cannot be converted into illicit drugs except through a series of complicated and inefficient chemical processes, which are assumed to be too complicated to be used to produce illegal drugs^8–10^. In contrast to opium poppy, where morphine is the main alkaloid accumulated in capsules, thebaine is the dominant alkaloid that is synthetized in different tissues and occurs in higher level in roots and capsules in Persian poppy, *Papaver bracteatum* Lindl^11–14^. Therefore, *P. bracteatum* is a good substitute for farmers to replace opium poppy by the legally much less problematic Persian poppy, as an increasing worldwide demand of its alkaloids arose over the last decades to be used by the pharmaceutical industry as an alternative to traditional opium poppy^15^.

*Papaver bracteatum* (2*n* = 2*x* = 14 chromosomes) is a perennial species that along with the polyploid species *P. orientale* L. (2*n* = 4*x* = 28) and *P. pseudo-orientale* (Fedde) Medw. (2*n* = 6*x* = 42) constitute *Papaver* section *Oxytona* Bernh^16,17^. *Papaver bracteatum* is distributed at elevations mainly between 1500 and 2500 m with its main occurrence areas in the Zagros Mountains in west and northwestern Iran, the Alborz Mountains in the north of Iran, and the southern parts of the Caucasus in northwestern Iran, Georgia and Azerbaijan, and northeastern Turkey^8,14,17^. In the north its distribution area is overlapping with the two polyploid species of the section and hybrids seem to occur^16^.

**Figure 1:**
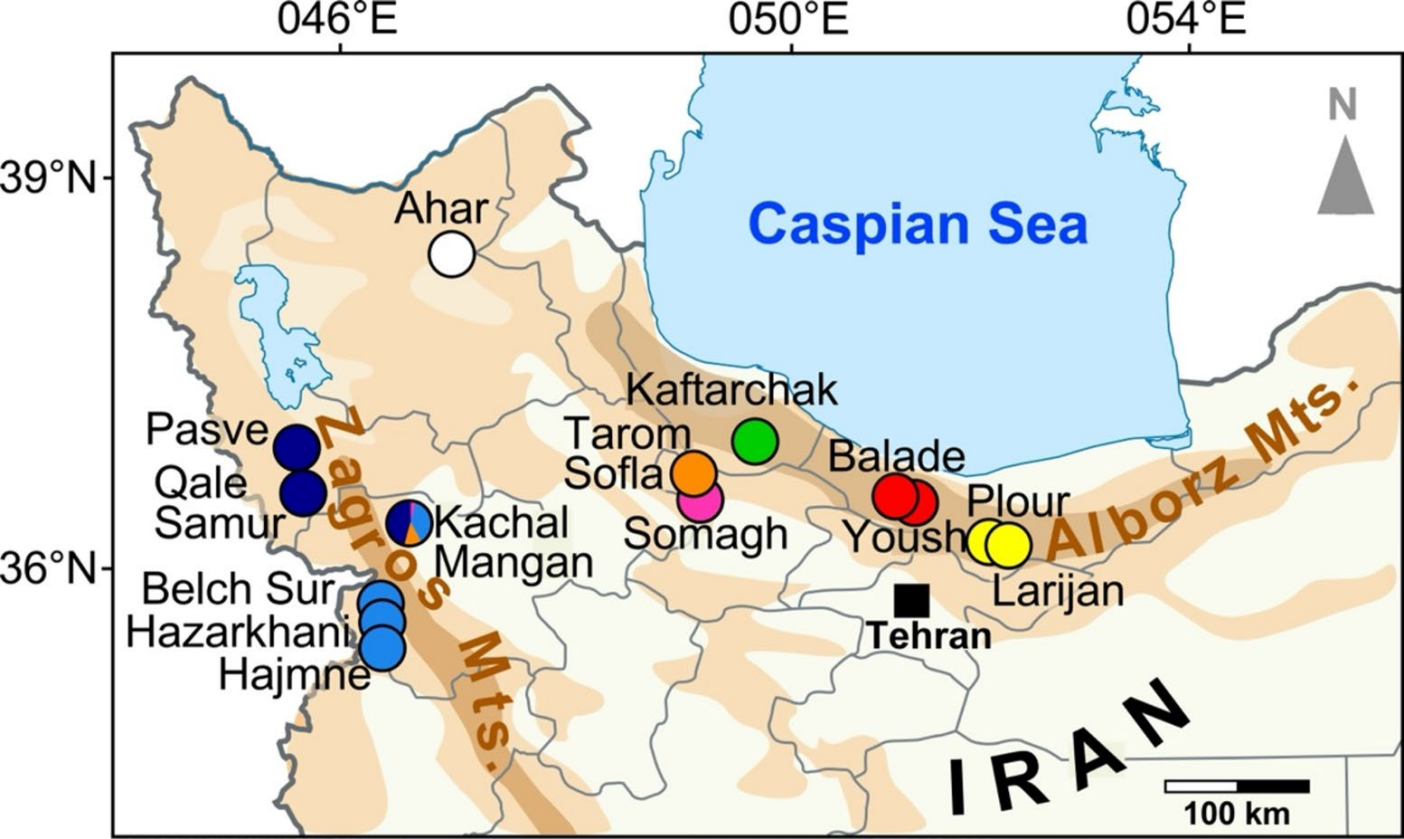
Map of northwestern Iran providing the geographic positions of *P. bracteatum* and *P. orientale* populations included in the analyses. Dot colors refer to population assignment analysis (at K = 7) for *P. bracteatum*, while the white dot marks the origin of the *P. orientale* population.

The knowledge of genetic variation and population structure of the species can contribute to germplasm conservation and breeding of the plant. Despite medicinal importance of *P. bracteatum*, there is no comprehensive study inferring genetic diversity and genetic structure of *P. bracteatum* populations, mainly due to lack of enough DNA polymorphisms found in the up to now conducted studies^18,19^. Genotyping-by-sequencing (GBS) is one of the most efficient approaches for the study of populations and closely related species complexes, as it detects genome-wide single-nucleotide polymorphisms (SNPs) and allows their use for genotyping^20–22^. Here, we employed such SNP markers obtained through GBS for individuals from 13 natural populations of *P. bracteatum* in Iran (Fig. 1 and Table 1). As this area covers the center of distribution of the species, our diversity panel covers the natural diversity of the species in Iran and most probably is representative for the entire species. The specific goals of this study are: (1) to infer phylogegraphic relationships, (2) to resolve population structure into different gene pools, (3) to analyze gene flow and admixture between intraspecific gene pools, and (4) to test the impact of environment on gene flow for *P. bracteatum* populations. From this we want to arrive at a better understanding of the genetic characteristics of *P. bracteatum*, which could underpin future utilization and protection of the genetic resources of Persian poppy.

**Table 1:** Populations included in the analyses.

Species	Population (abbreviation)	Origin	Coordinates	Elevation (m)	No. of individuals
*Papaver orientale*	Ahar	Iran, Province East Azerbaijan, close to Ahar	38° 21′ 52′′ N 47° 6′ 57′′ E	2105	20
*Papaver bracteatum*	Balade (Bal)	Iran, Province Mazandaran, around Balade, Alborz Mts.	36° 14′ 13′′ N 51° 26′ 24′′ E	3041	19
	Belch Sur (BSr)	Iran, Province Kurdistan, near Belch Sur, Zagros Mts.	35° 22′ 45′′ N 46° 23′ 16′′ E	2714	14
	Hajmne (Hjn)	Iran, Province Kurdistan, near Hajmne, Zagros Mts.	35° 22′ 50′′ N 46° 22′ 40′′ E	2431	30
	Hazarkhani (Hkn)	Iran, Province Kurdistan, near Hazarkhani, Zagros Mts.	35° 22′ 32′′ N 46° 22′ 52′′ E	2389	28
	Kachal Mangan (KMn)	Iran, Province Kurdistan, near Kachal Mangan, Zagros Mts.	36° 08′ 37′′ N 46° 20′ 11′′ E	1963	20
	Kaftarchak (Kck)	Iran, Province Gilan, near Kaftarchak, western Alborz Mts.	36° 42′ 45′′ N 49° 43′ 52′′ E	1879	20
	Larijan (Ljn)	Iran, Province Mazandaran, near Larijan, central Alborz Mts.	35° 51′ 4′′ N 52° 4′ 24′′ E	2364	15
	Pasve (Psv)	Iran, Province west Azerbaijan, near Pasve, Zagros Mts.	36° 44′ 53′′ N 45° 28′ 17′′ E	1856	13
	Plour (Plr)	Iran, Province Mazandaran, near Plour, central Alborz Mts.	35° 51′ 4′′ N 52° 4′ 24′′ E	2369	15
	Qale Samur (QSr)	Iran, Province West Azerbaijan, near Qale Samur, Zagros Mts.	36° 19′ 59′′ N 45° 34′ 57′′ E	1628	15
	Somagh (Sgh)	Iran, Province Qazvin, near Somagh, west Alborz Mts.	36° 21′ 45′′ N 49° 23′ 33′′ E	1946	15
	Tarom Sofla (TSl)	Iran, Province Qazvin, near Somagh, west Alborz Mts.	36° 37′ 14′′ N 49° 7′ 22′′ E	2072	20
	Yoush (Ysh)	Iran, Province Mazandaran, near Yoush, central Alborz Mts.	36° 13′ 52′′ N 51° 25′ 16′′ E	2719	20

## Results

### Ploidy level estimations

We used flow cytometry to infer the ploidy levels for the individuals of all analyzed populations, as this is a fast way to discern diploid *P. bracteatum* from the polyploids within sect. *Oxytona*. Genome size measurements against a pea reference resulted in a 2C genome size of 5.93 pg (SD ± 0.24) for *P. bracteatum* and 15.69 pg (SD ± 0.19) for the measured *P. orientale* individuals. Thus, all our analyzed populations of Persian poppy were diploid while the *P. orientale* values indicate a genome size that is higher than what would be expected for a tetraploid.

### Genotyping-by-sequencing

After removing the barcodes and quality filtering, our GBS data consisting of *P. bracteatum* together with *P. orientale* resulted in on average 5764 loci (min.: 3400, max.: 5923) with a concatenated length of the alignment of 659,080 nt with 3.87% missing sites. This dataset was used for the phylgeographic analyses. For the dataset consisting only of *P. bracteatum* individuals, we obtained an average of 11,690 loci (min.: 9152, max.: 11,978) per individual, with a concatenated alignment length of 1,306,446 nt with 3.60% missing sites. This dataset was employed for population assignment and genetic diversity analyses.

### Population structure

**Figure 2:**
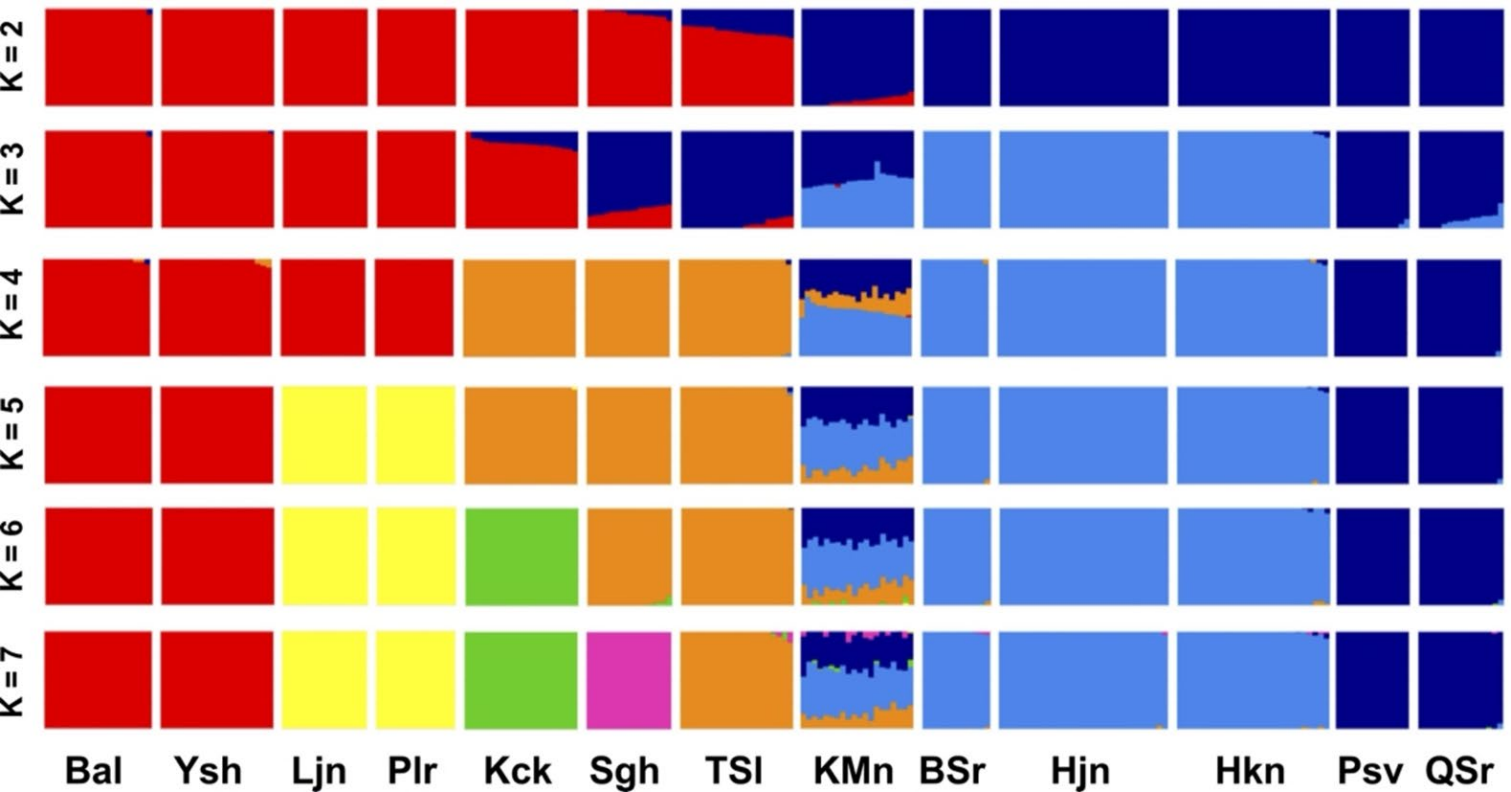
Population assignment analyses from K = 2 to K = 7 based on 6027 unlinked SNPs of 244 *P. bracteatum* individuals. Each box shows a population with the containing individuals represented by thin bar plots. The colors indicate the assignment of the individuals to K populations.

The Bayesian population assignment analyses using 6027 unlinked high-quality SNPs in *P. bracteatum* provide information for the allelic patterns of the populations and admixture patterns between individuals (Fig. 2). At K = 2 populations are divided into two subgroups representing Alborz Mts. versus Zagros Mts. populations with admixture signals for the populations in between the two mountain systems (Kachal Mangan, Somagh, Tarom Sofla). At K = 3 the northern (Pasve, Qale Samur) and southern (Belch Sur, Hajmne, Harzakhani) Zagros Mts. populations split. At K = 4 the geographically intermediate populations with an admixture signal in the analysis before, now together with Kaftarchak, form a group of their own. All Kachal Mangan individuals show strong admixture, a signal that is maintained through all steps up to K = 7. With further increasing K the Alborz Mts. populations split, first the easternmost group, then the populations in the west of the Alborz Mts. system. Thus, at K = 7 the 13 analyzed populations are grouped in seven allelic groups plus one with a strong admixture signal. The geographic position of the latter (Kachal Mangan) is the easternmost in the Zagros Mts. and it occupies a kind of intermediate position between both mountain systems (Fig. 1), which correlates with its mixed allelic constitution in this analysis: Alleles are mainly shared with the Zagros Mts. populations but also with Tarom Sofla and, to a small extend, Somagh.

**Figure 3:**
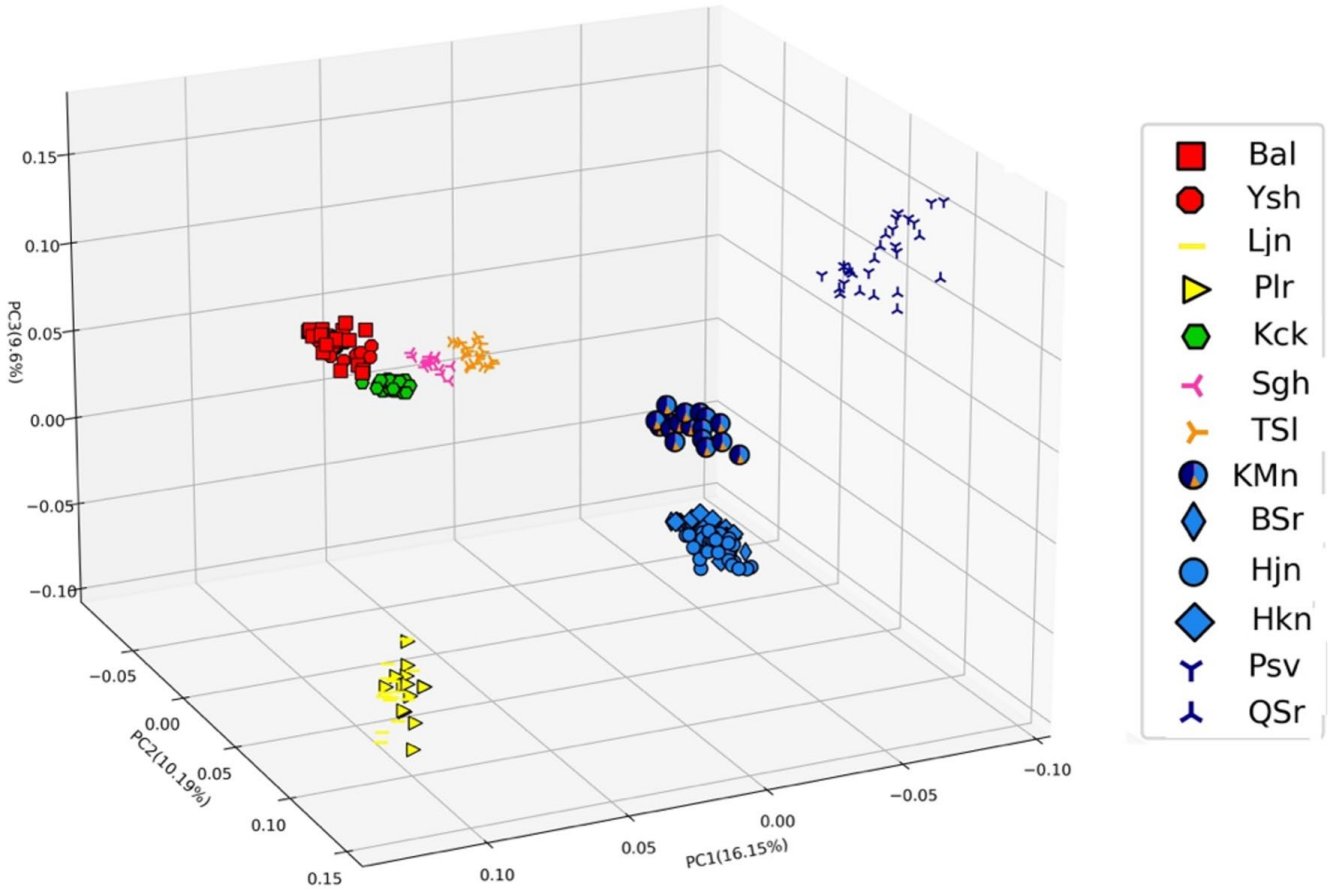
Plot of the first three axes of a Principle Component Analysis based on 6027 genome-wide unlinked SNPs scored in 244 *P. bracteatum* individuals.

In our PCA the first two principal components explained 26.3% of the total molecular variation. The results (Fig. 3) are consistent with the admixture plots at K = 5 by separating the populations along their geographical origins and placing Kachal Mangan individuals in a central position.

F_ST_ values were used to quantify the level of genetic structure among populations. The pairwise F_ST_ value-derived differentiation between 13 populations of *P. bracteatum* (Supplementary Table S1) ranges from very little between populations within the Zagros Mts., i.e. Hajmne versus Belch Sur and Hazarkhani (0.026), to very strong between the geographically most distant populations, i.e. Pasve versus Plour and Larijan (0.26). The average F_ST_ value is equal to 0.16, indicating high differentiation among populations.

Since our analyses show individuals from Kachal Mangan as admixed (Fig. 2), another pairwise F_ST_ comparison was conducted to measure differentiation between these individuals and populations from other subgroups. The pairwise F_ST_ reveals lower differentiation of Kachal Mangan towards populations Tarom Sofla (0.118), Belch Sur/Hajmne/Hazarkhani (0.056) and Pasve/Qale Samur (0.089) in comparison to the remaining combinations (> 0.15; Supplementary Table S2) concurring the admixed nature of the Kachal Mangan population.

### Genetic diversity analyses

Genetic diversity analyses were conducted to elucidate the genetic landscape of *P. bracteatum* populations, focusing on observed heterozygosity, nucleotide diversity (π), and the inbreeding coefficient (F).

**Figure 4:**
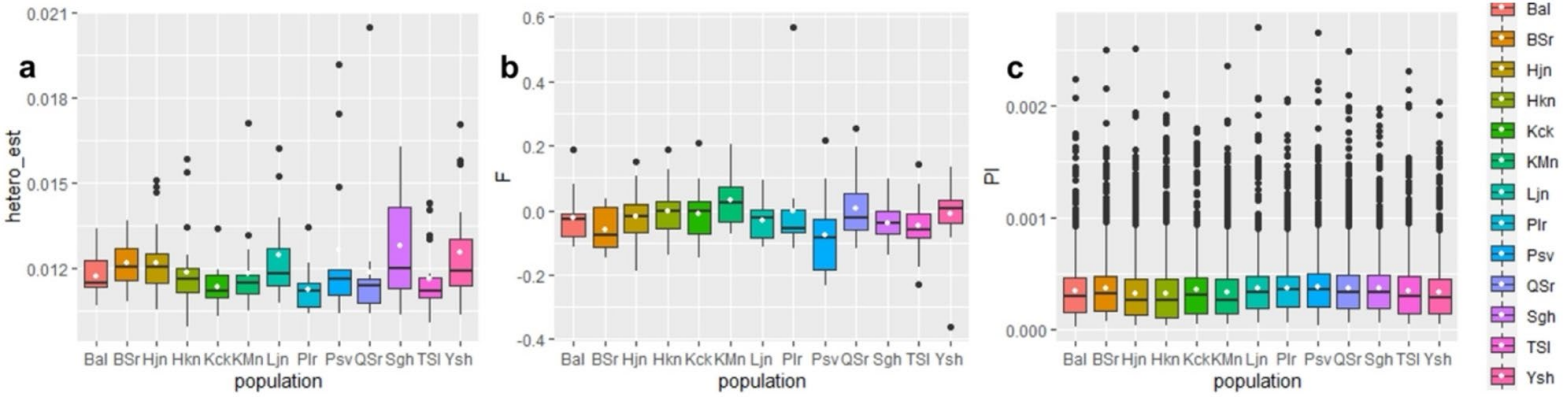
Panel (**a**) shows individual observed heterozygosity across loci within each population. Panel (**b**) illustrates the inbreeding coefficient for individuals in each population. Panel (**c**) presents the distribution of nucleotide diversity for each population. Median values are shown by thick black lines, with boxes indicating quartiles. Whiskers extend to 1.5 times the interquartile range or the maximum value. White circles denote the mean value of each parameter for each population, while the black circles show outliers.

Observed heterozygosity, reflecting the presence of diverse alleles within a population, was assessed across populations (Fig. 4a). Notably, populations from Somagh in the western Alborz Mts., as well as Yoush and Larijan in the central Alborz Mts., exhibited higher levels of heterozygosity compared to other populations, including the admixed population of Kachal Mangan from Kurdistan province in the Zagros Mts.

Variation in the inbreeding coefficient was observed among populations, with elevated levels detected in Kachal Mangan, followed by Qale Samur, compared to other populations. Conversely, Pasve, located in the northwest of Iran in the Zagros Mts., demonstrated the lowest level of inbreeding coefficient (Fig. 4b). Nucleotide diversity analysis revealed a relatively modest level of genetic variation at the nucleotide level within *P. bracteatum* populations, with values ranging from 0.318 × 10^–3^ to 0.382 × 10^–3^ (Fig. 4c).

### Phylogenetic inferences

**Figure 5:**
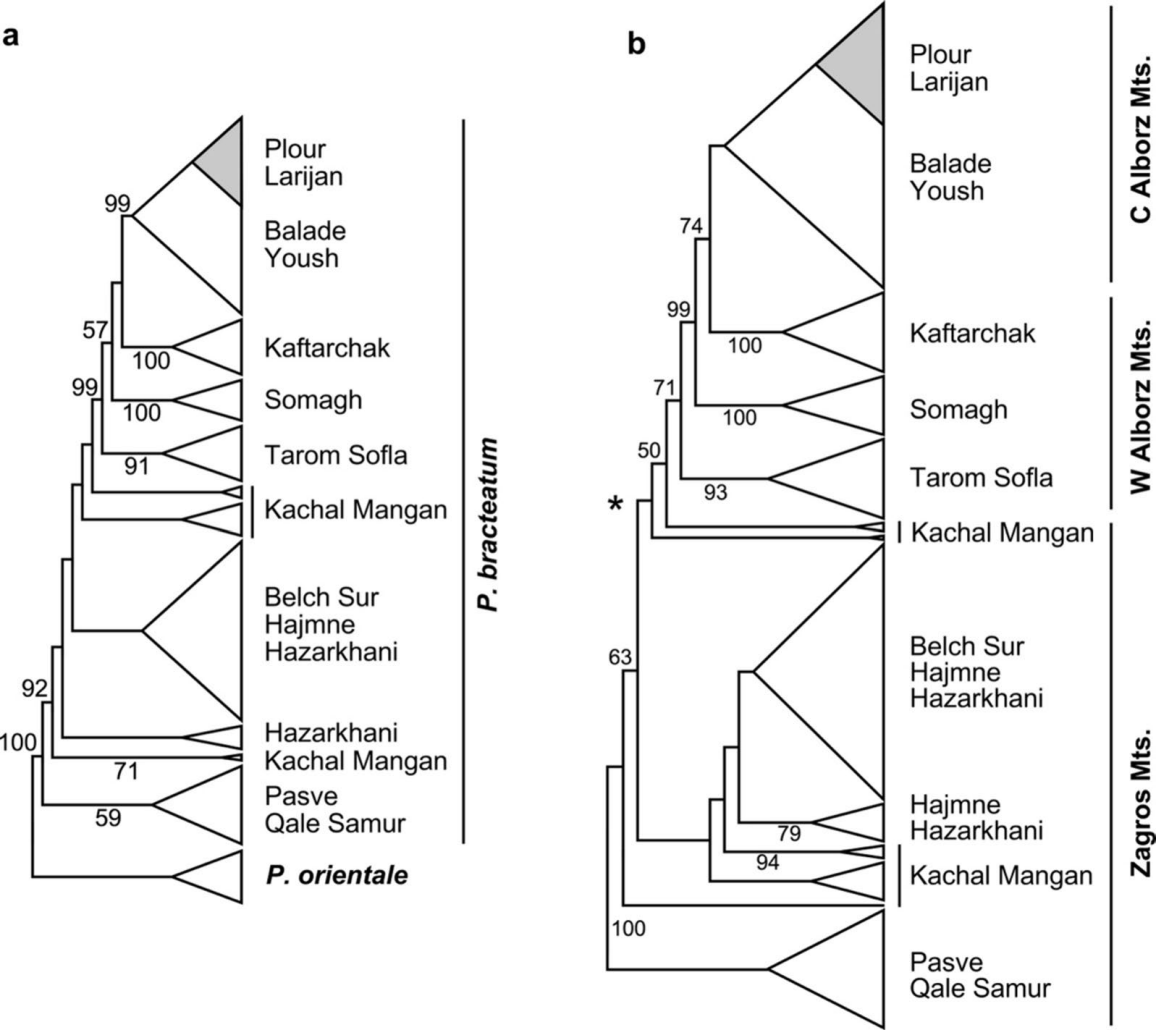
(**a**) Schematic representation of the strict consensus tree topology of 80 most parsimonious trees derived from an MP analysis of the GBS dataset including 264 individuals of *P. bracteatum* and *P. orientale*. Numbers along the branches provide bootstrap support values (%) for the tree backbone, names to the right the population affiliations for *P. bracteatum*. The tree was rooted with the *P. orientale* individuals. (**b**) Schematic representation of the strict consensus tree topology of 120 MP trees derived from the analysis of GBS data of 244 individuals of *P. bracteatum*. The tree was rooted with the Pasve/Qale Samur individuals to make it easier comparable with Fig. 5a. An asterisk marks the position of the root according to a population assignment analysis (Fig. 2), which separates at K = 2 the populations from Zagros versus Alborz Mts. Numbers along branches provide bootstrap support values (%) for the tree backbone. Names to the right indicate population affiliations. The individuals from Larijan and Plour form in both trees a monophyletic unit within the polyphyletic populations from Balade and Yoush. Also the individuals from Kachal Mangan result in both analyses polyphyletic, indicating their mixed ancestry.

We used maximum parsimony (MP) and maximum likelihood (ML) algorithms to infer population relationships according to our GBS data. The MP analysis of the dataset including *P. orientale* as outgroup was based on the alignment of 659,080 nt length. It had 5892 parsimony-informative characters and resulted in 80 equally parsimonious trees of 28,095 steps length with a consistency index (CI) of 0.30 and a retention index (RI) of 0.67. For the MP analysis of *P. bracteatum* we used the same alignment but excluded the 20 *P. orientale* individuals from the dataset. This dataset had 2979 parsimony-informative characters and resulted in 120 equally parsimonious trees with a length of 21,890 steps (CI = 0.20, RI = 0.56). Schematic representations of the strict consensus trees of both analyses are provided in Fig. 5, the detailed consensus trees as Supplementary Figs. S2 and S3.

The initial analysis, where we included *P. orientale* individuals from Ahar, showed that these tetraploids have the highest similarity within *P. bracteatum* in the geographically closest populations in the north of the Zagros Mts. This results in the Pasve/Qale Samur populations occupying the position as sister group to all other *P. bracteatum* individuals in the MP tree (Fig. 5a). However, with our sampling design including *P. orientale* from just one area and not separating homoeologous alleles in the tetraploid, we cannot conclude that this is evolutionary correct. This means that a biogeographical interpretation of the trees, which would imply that *P. bracteatum* originated in the Zagros Mts. and afterwards colonized the Alborz Mts. from west to east, is not possible based on this dataset. Thus, we analyzed the dataset in addition without the *P. orientale* individuals. This MP analysis was essentially an unrooted analysis where the tree was afterwards drawn to make it easily comparable with the result of the analysis including *P. orientale* (Fig. 5b). With midpoint rooting or putting the root according to the result of the population assignment analyses, the Zagros and Alborz groups would be sisters, with the Kachal Mangan population at an intermediate position. This topology is indicated by an asterisk at the root position in Fig. 5b.

Within *P. bracteatum* we found in both analyses geographically close populations grouping together in the phylogenetic trees. In some cases, individuals collected from different populations like Pasve/Qale Samur or Balade/Yoush, resulted also in the phylogenetic trees intermingled and not separated according to their collection sites (Fig. 5 and Supplementary Fig. S3). This indicates close relationships of populations from within the two mountain systems. The Kachal Mangan population in the east of the Zagros Mts. combines alleles from both main groups and accordingly occupies an intermediate position. The hybrid nature of this population (Fig. 2) resulted in very low bootstrap support values for this group and also for the branches around their individual positions in the trees. Within the Alborz Mts. populations a geographic progression from west to east can be derived from this dataset (Figs. 1 and 5).

The main differences of the ML analysis of *P. bracteatum* towards the results of the MP analysis concerns (1) the position of the Kachal Mangan individuals, which in ML (Supplementary Fig. S4) form a grade at the base of the Alborz Mts. populations, while in MP Kachal Mangan individuals form grades at the base of the Alborz and Zagros Mts. populations (Fig. 5). In both cases, bootstrap support values for Kachal Mangan and the clades around are rather low, which is indicative of the hybrid nature of this population. (2) The second difference can be found within the group of central Alborz Mts. populations where Balade/Yoush and Larijan/Plour are sister groups in ML instead of the latter grouping within a grade of Balade/Yoush individuals in MP. Still, relationships among the populations reflect also in ML clearly the results of the population structure analyses.

### Ecoclimatic niche modeling

**Figure 6:**
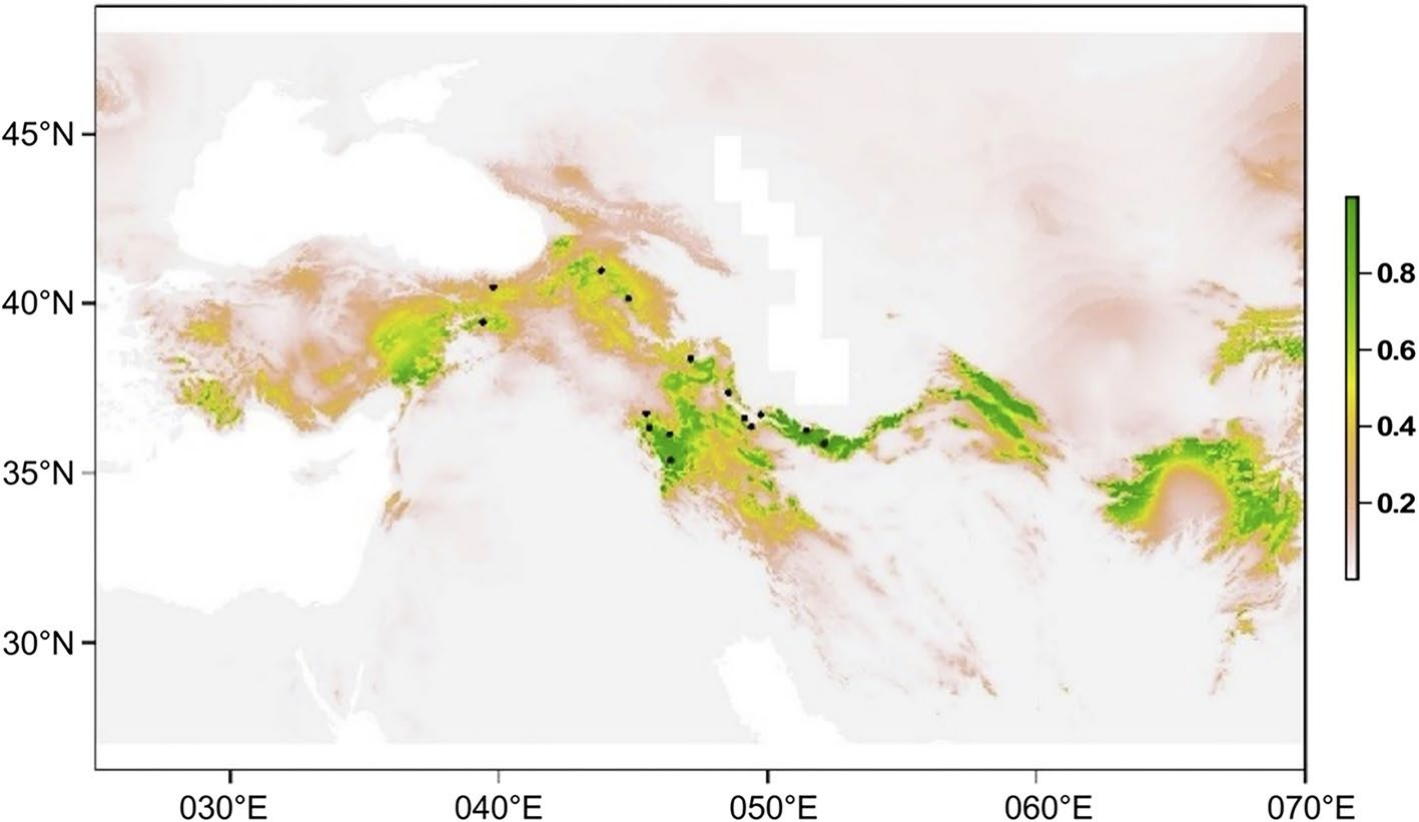
Ecoclimatic niche modelling for *P. bracteatum*. Black dots indicate the occurrence records, covering the entire distribution of the species, that were used as input for the niche models. The scale for habitat suitability is provided to the right. The niche models concur very well with the actual distribution of the species in northeastern Turkey and Iran. For southeastern Turkey and parts of Central Asia, however, they show that the potential distribution area/climate-defined niche of *P. bracteatum* is much larger than the regions currently inhabited by the species.

The best potential distribution-envelop model was obtained using the following settings: regularization value 1 and set of tested feature classes LQH, respectively. Figure 6 shows the resulting map of our ecoclimatic niche modeling approach. Suitable ecoclimatic conditions were found in the mountain ranges from the eastern part of the Taurus in Turkey, the Armenian Highlands, the northern part of Zagros Mts., Alborz to Zagros Mts. and extended eastwards to the mountain systems of Central Asia (Hindukush and Himalayas). Thus, our prediction of areas with possible suitable climate conditions for *P. bracteatum* are much larger than the currently realized distribution area of the species, with absence or very rare occurrences of the species in Central Asia and southwestern Turkey.

### Modeling population demographic history

**Figure 7:**
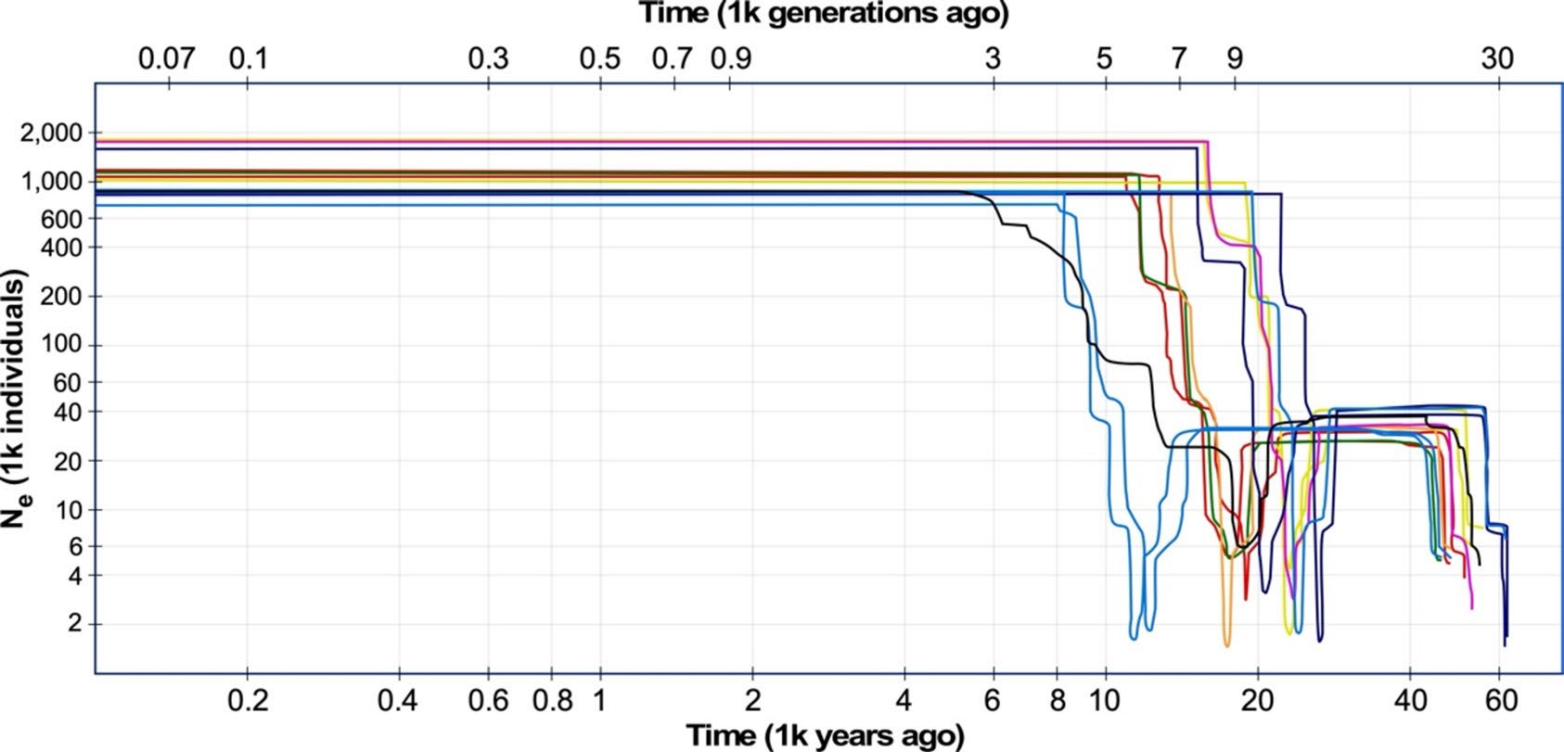
Composite plot of demographic changes within the analyzed *P. bracteatum* populations during the last 50,000 years. Colors indicate the populations as given in Fig. 1. For detailed graphs of the single populations including confidence intervals see Supplementary Fig. S5. All populations share a signal for a genetic bottleneck between the Last Glacial Maximum (~ 18,000 years ago) and the onset of Holocene when temperatures started to rise to their extant level.

The results of demographic modeling show a genetic bottleneck for all populations when effective populations sizes (N_e_) decreased substantially, but only for a short time period, at the end of the Pleistocene (Fig. 7 and Supplementary Fig. S5). However, the exact timing varied according to population, with the time estimates for the decrease ranging from around the Last Glacial Maximum to the beginning of the Holocene. After recovery, which happened rather quickly in most populations, N_e_ remained stable in all examined populations until today. Demographic modeling did not provide evidence for recent declines in N_e_. In general, N_e_ of populations were lower during the last glacial period than today.

## Discussion

*Papaver bracteatum* is known as a potential source of raw material for the production of codeine, without the involvement of morphine. Despite pharmaceutical importance of *P. bracteatum*, genetic variation and genetic structure of natural populations are still unclear. Hence, to investigate phylogeography and population structure of the species in Iran, we here used genome-wide distributed SNP markers obtained with high-throughput genotyping-by-sequencing (GBS) on accessions belonging to 13 natural populations of *P. bracteatum* and one population of its closely related species *P. orientale*.

Based on our result, the 13 populations were clustered into seven subgroups: (1) Larijan and Plour, (2) Balade and Yoush, both subgroups occurring in the central Alborz Mts. (Fig. 1), (3) Kaftarchack, (4) Somagh and (5) Tarom Sofla, all from western parts of the Alborz Mts., (6) Belch Sur, Hajmne and Hazarkhani from Kurdistan province in the Zagros Mts., and (7) Pasve and Qale Samur from Azerbaijan province in the northern Zagros Mts. The successive subdivisions of these populations in population-assignment groups from K = 2 to K = 7 (Fig. 2) is reflected in the clades that were obtained by phylogenetic analyses (Fig. 5) when midpoint rooting of the tree is applied. We found a clear geographic pattern within Iranian *P. bracteatum* populations with close relationships among the Zagros Mts. and the Alborz Mts. populations, respectively. Within the Alborz Mts. we can deduce also a colonization pattern from west to east, as the easternmost populations are nested within a grade of populations from further west. For the populations from within the Zagros Mts. a colonization pattern is not obvious. All these populations show at K = 7 in our population assignment analysis very low signals of admixture.

In contrast to the populations mentioned above, the Kachal Mangan population from the east of Zagros Mts. that geographically is in an intermediate position between the western and eastern populations (Fig. 1), was obtained as admixed in all analyses from K = 4 to K = 7. Also in phylogenetic analyses, this population showed all signs of pronounced admixture, with (1) an intermediate position between western and eastern groups in the trees, (2) low bootstrap support values in all analyses for its position but also for the branches that connect its subgroups to other populations, and (3) non-monophyly for the Kachal Mangan individuals forming subclades that are sister to either Zagros or Alborz populations, depending on the allelic composition of the individuals. Analysis of F_ST_ values (Supplementary Tables S1 and S2) and PCA (Fig. 3) support this intermediate position of the Kachal Mangan individuals with alleles shared with its neighboring populations east and west. This pattern could either be interpreted as the Kachal Mangan area being the center of origin of the species and still maintaining a diverse set of ancient alleles, which got sorted and partly lost when the species spread east- and westwards out of this area. Alternatively, Kachal Mangan might be a meeting point where eastern and western populations came into contact, resulting in introgression and accordingly admixed allelic signals for its individuals. Genetic distances and heterozygosity within the Kachal Mangan population are not larger than in the other populations around (Fig. 4), which might indicate that it is not an ancient population but a Zagros population that was introgressed sometime in the past by Alborz Mts.-derived gene flow. This population is also different regarding its demographic recovery after the genetic bottleneck all populations experienced at Last Glacial Maximum and before the onset of the Holocene (Fig. 7, Supplementary Fig. S5). In contrast to the other populations, its N_e_ increased here stepwise over a longer time period, which might be attributed to repeated immigration events from western and eastern genotypes. It will be interesting to see if the strong genetic bottleneck at the end of the Pleistocene is a peculiarity of *P. bracteatum* populations or a feature that might be shared by other taxa from the Irano-Turanian floristic area.

In contrast to our result, Qaderi et al.^18^ using inter simple-sequence repeats (ISSR) and start-codon targeted (SCoT) markers clustered six populations of *P. bracteatum* into three distinct subgroups without any signal of admixture. Furthermore, we here observed a relatively high fixation index (F_ST_ = 0.16) as a measure of genetic differentiation among *P. bracteatum* populations, which is still low in comparison with previous studies of Persian poppy presented so far (Hadipour et al.^19^: G_ST_ = 0.52; Qaderi et al.^18^: G_ST_ = 0.53). This suggests a relatively low level of gene flow and clear genetic differentiation among populations from different geographical regions. The resolving power of markers in the inference of phylogenetic relatedness and evolutionary events is mainly determined by the level of polymorphism detectable. Therefore, the higher differentiation observed among populations in the previous studies can be attributed to the markers used, where fast-evolving marker regions like microsatellite-targeting ISSRs show probably less shared alleles in comparison to more slowly evolving ones like GBS. In the latter, due to using the methylation-sensitive enzyme *Msp*I, SNPs are mainly detected in the under-methylated (i.e. functional/coding) genomic parts, which generally evolve slower than non-coding fractions of the genome. Moreover, GBS arrives at a large number of genome-wide distributed SNP markers within *P. bracteatum*, which might not be comparable to the limited number of informative genomic areas probed by methods like ISSR, amplified fragment length polymorphisms (AFLP) and SCoT in previous studies^18,19^.

In *P. bracteatum* different mechanisms influence gene flow and population connectivity. On the one hand, these are the abilities of the plants to disperse pollen and seeds, which is influenced not only by the kind of pollinators and morphological seed structures that support seed transportation but also by geographic settings in the species’ occurrence areas. The widely open *Papaver* flowers are unspecialized regarding their pollinators^23^, which often are beetles, flies and wild bees. This means that pollen is mostly transported in a range of a few meters up to a few hundred meters^24^. Also, seed dispersal in *Papaver* is mostly restricted to distances of centimeters to few meters^25^, but might increase when seeds are transported by water after shedding from the capsule. However, obligate outcrossing of *P. bracteatum*^17^ will restrict rare water- or wind-mediated long-distance dispersals, as single plants that are established away from the nearest population will not set seeds due to their inability of selfing. In addition, the adaptation of *P. bracteatum* to mountain habitats might have resulted in population contacts in lowlands during glacial cold cycles in the Pleistocene but prevents the colonization of intervening lowlands during warm inter-glacial periods and under current climate conditions. Thus, the differentiation we see today might be influenced by population contacts during glacial maxima, resulting in gene flow and admixture where the different genotypes had met. Under extant climate conditions *P. bracteatum* populations are clearly isolated, a tendency that will increase with climate warming. This notion is supported by the predicted potential distribution area of the species (Fig. 6) that is much larger than the currently realized distribution. This could be attributed to relatively low colonization abilities of the species, resulting in slow migration into suitable areas if far away from extant populations. In addition, human impact on the habitats of the already fragmented populations of *P. bracteatum* will increase pressure on the species^26–28^. Sensitivity of the species towards changing environmental conditions can also be deduced from the demographic histories of the species, where a sharp decline of effective population size at the end of the Late Pleistocene was found (Fig. 7 and Supplementary Fig. S5). The population decline may be linked to abruptly changing climatic conditions at the end of the last glacial period. As temperatures rose very quickly^29,30^, populations may not have been able to migrate fast enough to track suitable habitats and/or adapt to the new environmental conditions. The stability in population sizes from the time of recovery towards today indicates that the much less marked climatic changes in West Asia during the Holocene^31^ did not leave demographic footprints. However, the natural habitat of *P. bracteatum* are rocky slopes and grassy meadows at elevations mainly between 2000 and 2500 m^13^, which during the last decades got in Iran under increasing pressure from livestock grazing. Overgrazing not only affects the *P. bracteatum* populations directly but entire natural habitats, as it significantly reduces the vegetation cover, which leads to the decline in pollinator activity in these areas. Consequently, the chances of inter-population pollination get more limited, and isolated, genetically more homogeneous populations are expected.

We studied only the populations from the center of distribution of *P. bracteatum*. To see how representative our data are for the entire species, it would be of interest to include now also populations from its more peripheral occurrence areas in eastern Turkey, Armenia and also from highlands east of the Alborz Mts. Although population numbers are rapidly declining with distance from the center, it would add to the general understanding of the population dynamics and genotypic diversity of *P. bracteatum* and improve knowledge of colonization patterns in the Irano-Turanian floristic region^32^.

Due to low gene flow and high genetic variation between *P. bracteatum* populations, future conservation programs for this species should include representative populations of the diverse genotype groups we describe here to arrive at the highest genetic variation for both seed and germplasm conservation. We show that our GBS-based approach is able to infer the phylogeography and the population structure of *P. bracteatum* and is able to reliably distinguish the genetic variation in highly homogenous plant populations. The large number of genome-wide distributed SNP markers allows utilizing accessions for future conservation and improvement programs of *P. bracteatum.* It also shows that it is an important tool to direct conservation measures for other endangered species.

## Conclusions

This study provides comprehensive insights into the genetic diversity and unique population dynamics of *P. bracteatum* in Iran. A high-resolution phylogeny obtained by using GBS-derived SNP markers showed that Iranian *P. bracteatum* populations consist of seven genetic groups with a major split between the populations of the Alborz and the Zagros Mountain systems. Within these areas, five and two subgroups exist, respectively. One population from the eastern Zagros Mts. showed clear signals of introgression from its neighboring Zagros and Alborz populations, while otherwise, the populations seem clearly separated. Modelling population demography indicated strong genetic bottlenecks for all analyzed populations at the end of the last ice-age cycle when climate conditions rapidly changed. The species’ realized ecoclimatic niche is currently much smaller than its potential niche, indicating slow migration and niche filling. The low dispersal abilities of the species together with increasing habitat fragmentation due to human impact on the populations plus the current fast climate warming might warrant in- and ex-situ conservation measures for this valuable medicinal plant.

## Materials and methods

### Plant materials, DNA extraction and genome size measurement

We included 264 individuals consisting of 244 *P. bracteatum* and 20 *P. orientale* samples in the phylogenetic analyses (Table 1). The materials were collected as leaves and dried in silica gel in May 2021 in mountain steppe habitats throughout the distribution area of the species in Iran. *Papaver bracteatum* accessions were assigned to thirteen natural populations. The sampling sites (Fig. 1) were in the central Alborz Mts. of Balade (Bal), Yoush (Ysh), Larijan (Ljn) and Plour (Plr), west of the Alborz Mts. with the regions Kaftarchak (Kck), Somagh (Sgh), Tarom Sofla (TSl), and the Zagros Mts. regions of Kachal Mangan (KMn), Pasve (Psv), Qale Samur (QSr), Belch Sur (BSr), Hajmne (Hjn) and Hazarkhani (Hkn). Population samples were obtained through transects with about 20 m distance between the collected individuals. Genomic DNA was extracted from the dried leaves using the DNeasy Plant mini kit (QIAGEN) according to the instructions of the manufacturer. DNA quality was checked on agarose gels and for quantification the Qubit dsDNA High Sensitivity assay kit (Thermo Fisher Scientific) was used.

To confirm the uniformity of ploidy level of the analyzed accessions, genome sizes of one individual of each population of *P. bracteatum* and three individuals of *P. orientale* were measured using propidium iodide (PI) as a stain in flow cytometry with a Cyflow Space (Sysmex Partec) flow cytometer, following the procedure described by Jakob et al.^33^ The pea cultivar *Pisum sativum* L. ‘Viktoria’ (IPK gene bank accession number PIS 630; 10.25642/IPK/GBIS/27322) was used as the internal size standard (2C DNA content = 9.09 pg) in all measurements.

### Genotyping-by-sequencing

To obtain genome-wide SNPs, GBS analyses^20^ were used for 244 *P. bracteatum* and 20 *P. orientale* individuals. For assessment of reproducibility, four replicates of one sample were included in GBS. To prepare the library for each sample, 200 ng of genomic DNA was digested using two restriction enzymes, *Pst*I-HF (NEB) and methylation-sensitive *Msp*I (NEB). Library preparation, individual barcoding and 110 bp single-end sequencing on the Illumina NovaSeq were performed following Wendler et al.^34^ Library preparation and sequencing were conducted in the sequencing lab of the Leibniz Institute of Plant Genetics and Crop Plant Research (IPK), Germany.

The quality of raw fastq data was assessed using the fastqc toolkit^35^. Adapter trimming of GBS sequence reads was conducted with Cutadapt^36^ within ipyrad v.0.9.56^37^. GBS reads were clustered in ipyrad. We conducted two runs of ipyrad resulting in two GBS alignments concatenating all filtered loci: (1) one with all individuals, including also *P. orientale*, and (2) excluding the latter and restricting the dataset to *P. bracteatum* individuals. We set the minimal number of samples to possess a certain locus to 90% of the individuals and the clustering threshold of reads within and between individuals to 0.85 and 0.90 in the first and second datasets, respectively. The default settings were used for the other parameters. After a first check through a neighbor-joining analysis showed that the GBS results were highly reproducible, the replicated individual was included just once for all downstream analyses. For GBS-based phylogenetic analyses, the first dataset was used whereas for population assignment analyses and principal component analysis (PCA), we used the second dataset.

### Population structure and genetic diversity analyses

Single-nucleotide polymorphisms obtained by the ipyrad pipeline were subjected to quality control using plink 1.9^38^ with the following QC parameters: minor allele frequency < 0.01, Hardy–Weinberg *p* value < 0.000001, individuals with missing genotypes > 0.1 and missing rate per SNP > 0.1^38^. Population assignment analysis was conducted using the model-based Bayesian clustering software in Admixture 1.3^39^. The number of ancestral populations (K) was tested for a range from 1 to 13. The optimal K was determined based on cross validation error curve and the results were graphically represented using the R package ggplot2^40^ for K = 2 to K = 13, with an optimal K = 7 for this dataset.

Principle component analysis (PCA) was conducted in plink 1.9^38^ and the results were visualized using a Python script (https://github.com/Siavash-cloud/3D-PCA-plot).

We used the seven genetic subgroups identified in the population assignment analysis to test for genetic differentiation. The program Arlequin v 3.5^41^ was used to calculate pairwise genetic distances (F_ST_) between populations according to Weir and Cockerham^42^. F_ST_ values were interpreted according to Del Carpio et al.^43^, where an F_ST_ of 0 shows no differentiation between populations and a value of 1 shows complete differentiation. Populations were assumed to have little differentiation when F_ST_ values were below 0.05, moderate differentiation with values between 0.05 and 0.15, strong differentiation for F_ST_ values between 0.15 and 0.25, and very strong differentiation for values higher than 0.25.

Observed heterozygosity, reflecting the diversity of alleles within a population, was generated individually for each member of the population, obtained from the output result of ipyrad. Individual inbreeding coefficients and nucleotide diversity were calculated by employing VCFtools v 0.1.16^44^. VCFtools calculates the inbreeding coefficient (F) per individual using the equation F = (O − E)/(N − E), where O is the observed number of homozygous sites, E is the expected number of homozygous sites (given population allele frequency), and N is the total number of genotyped loci. Nucleotide diversity (π) is the average pairwise difference between all possible pairs of individuals in each population. The distribution of each parameter was calculated in R version 4.0.3 and shown as boxplot using ggplot2 in R^40^.

### Phylogenetic inference

To infer the phylogenetic relationships based on the GBS data, maximum parsimony (MP) analyses were conducted in Paup* 4.0a169^45^, with initially including all 264 individuals and defining *P. orientale* as outgroup, followed by an analysis of the dataset including only *P. bracteatum* individuals. For both analyses the concatenated alignment length was 659,080 bp. We conducted two-step MP analyses^46^ with an initial heuristic search with 500 random-addition sequences (RAS) restricting the number of stored trees to 25 per repetition, TBR branch swapping and steepest descent not enforced. The resulting trees were then used as starting trees in a heuristic analysis with maxtree set to 10,000. Clade support was evaluated by 500 bootstrap re-samplings with settings as before but excluding the initial RAS step.

In addition, phylogenetic relationships of *P. bracteatum* accessions were inferred using maximum likelihood (ML) based on the concatenated alignment applying the GTRGAMMA model and 100 parsimony starting trees in RAxML v.8.2.12^47^. Statistical support was assessed via rapid bootstrapping with 500 replicates.

### Ecoclimatic niche modelling

To obtain a map of habitat suitability and model the ecoclimatic niche of *P. bracteatum*, a maximum entropy approach was employed, as implemented in Maxent 3.3.3k^48^. Occurrence records were based on sampling locations of plant material used for phylogeographic analyses. Additional records were drawn from GBIF (www.gbif.org) and checked for plausibility of geographic coordinates. For modelling, a total of 22 records were used. Current bioclimatic data were downloaded from the WorldClim database (version 2.1, climate data for 1970–2000)^49^. To avoid collinearity among bioclimatic layers, five layers were selected using hierarchical clustering. The used bioclimatic layers were annual mean temperature, mean diurnal range (the mean of the difference of maximum and minimum monthly temperatures), isothermality, temperature annual range and precipitation of coldest quarter. Pairwise Pearson's correlation coefficients between the selected bioclimatic layers were < 0.7. The ‘ENMeval’ R package^50^ was used to build and compare several ecoclimatic niche models across a range of different settings. Ten thousand background points were drawn randomly from the study region, which was set to 25°–70°E and 27°–48°N. Regularization values were 0.5, 1, 2, 3, 4; the sets of tested feature classes were L, LQ, H, LQH, LQHP, LQHPT; test data were partitioned using the “checkerboard1” setting; and the number of kfolds for evaluation was set to 4. The best model was selected based on ΔAICc values.

### Modelling population demographic history

Population demographic histories were reconstructed using Stairway Plot 2.1.2^51,52^, based on folded site frequency spectra generated with easySFS (https://github.com/isaacovercast/easySFS). As input for easySFS, the ipyrad vcf output was used, after previous filtering employing VCFtools 0.1.16 with the following settings: max-missing 0.9; min-meanDP 20; max-meanDP 100. For demographic modelling, mutation rate was set to 7 × 10^–9^ substitutions per site per generation^53^. The generation time of *P. bracteatum* was set to two years. Singletons were included in the analyses.

### Plant collections

All plant experiments complied with relevant institutional, national, and international guidelines and legislation.

### Supplementary Information

Supplementary Information.

## Data Availability

All sequence data are available through the NCBI nucleotide database under bio-project number PRJNA998183.
