# Supplementary material for: Phylogeography and genetic structure of Papaver bracteatum populations in Iran based on genotyping-by-sequencing (GBS)
Source: Sci Rep. 2024 Jul 15;14:16309. doi: 10.1038/s41598-024-67190-8 (PMC11251027; doi:10.1038/s41598-024-67190-8)
Supplement: Supplementary file 1 — Supplementary Information. [file 41598_2024_67190_MOESM1_ESM.pdf]

Phylogeography and genetic structure of *Papaver bracteatum* populations in Iran based on genotyping-by-sequencing (GBS)

Razieh Rahmati, Zahra Nemati, Mohammad Reza Naghavi, Simon Pfanzelt, Amir Rahimi, Ali Ghaderi Kanzagh, Frank R. Blattner

Supplementary Materials

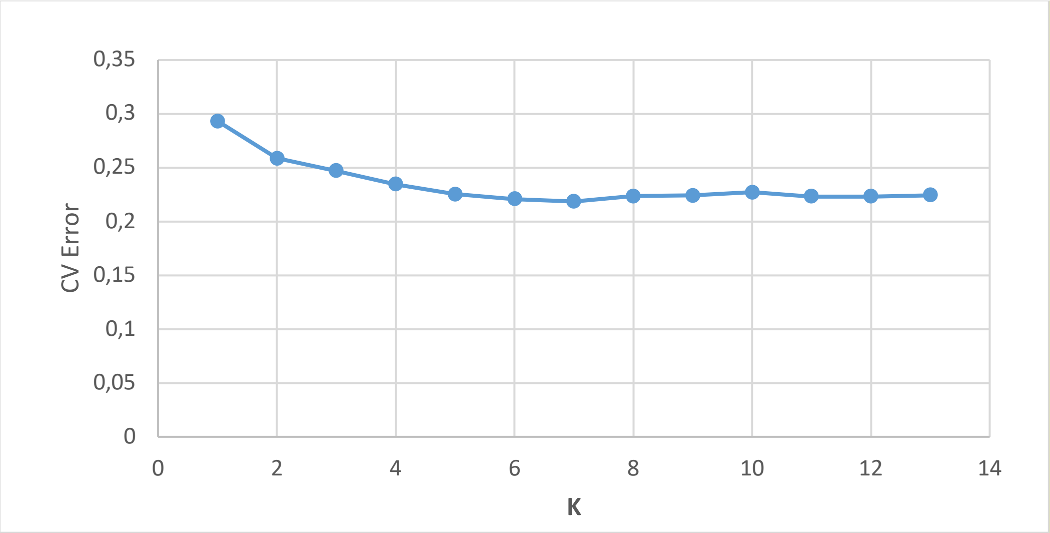

Figure S1. Cross-validation error curve for different K from 2 to 13 in population assignment analysis.

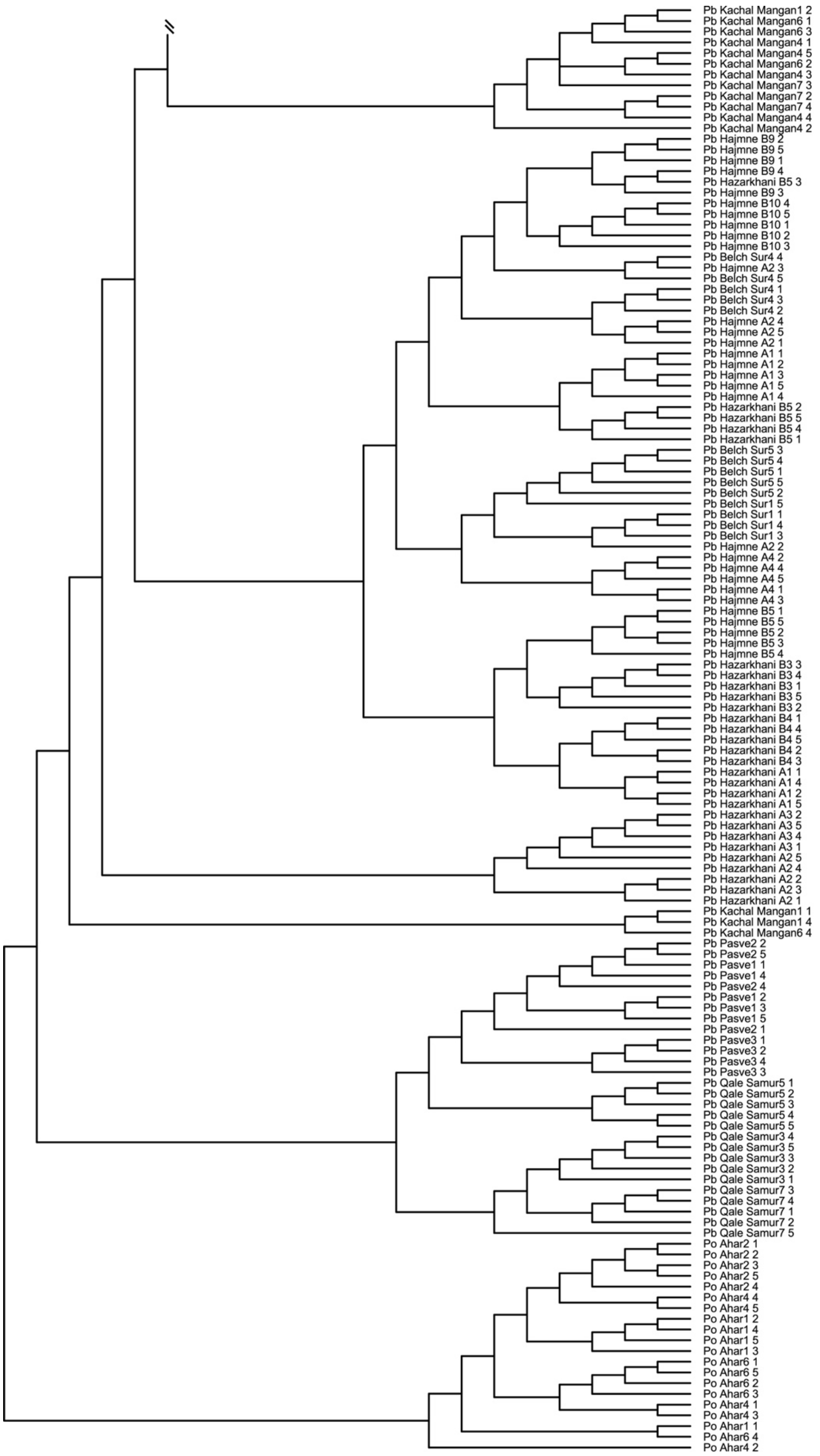

Figure S2. Strict consensus derived from 80 MP trees resulting from the parsimony analysis of the GBS dataset, including 264 individuals of *P. orientale* (Po; outgroup) and *P. bracteatum* (Pb).

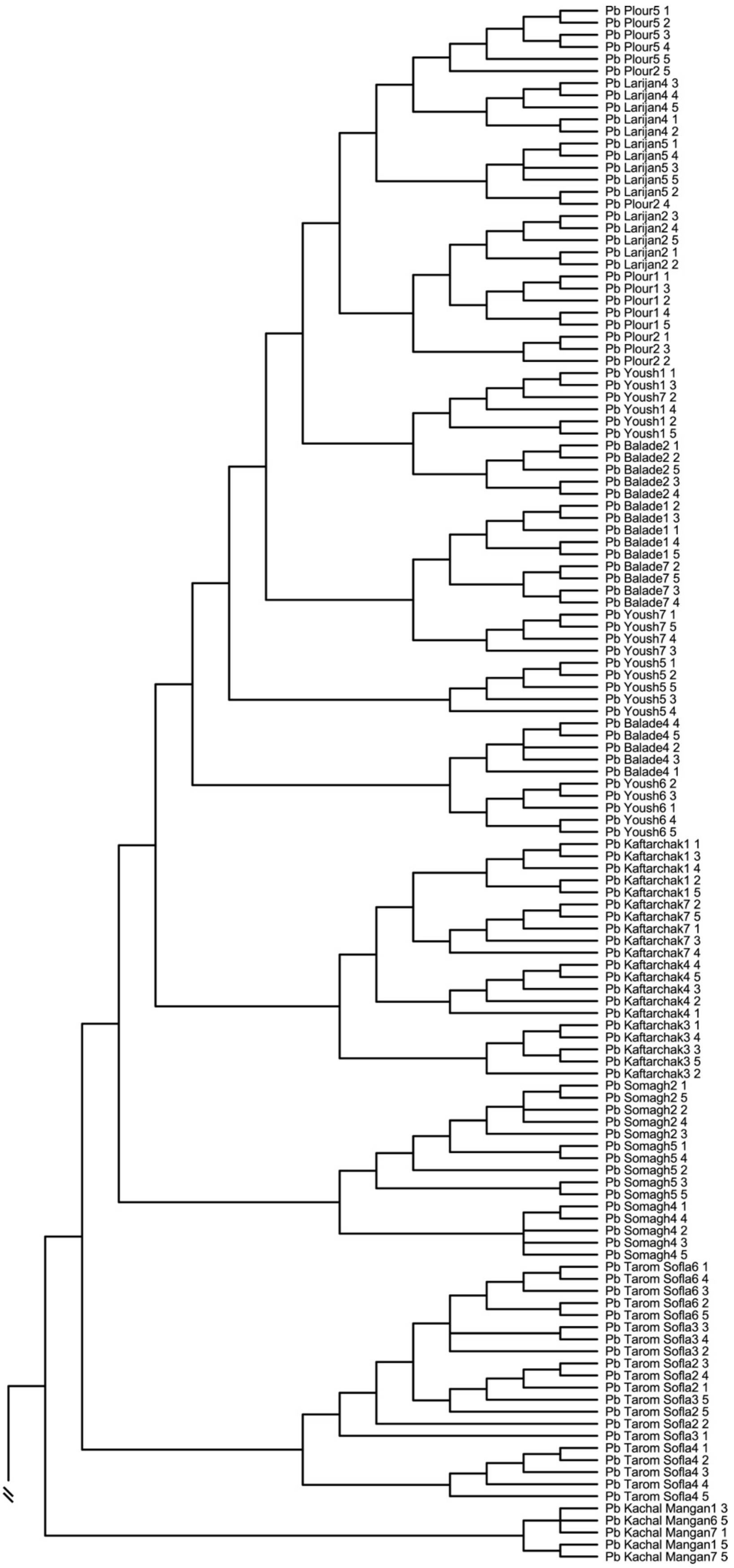

Figure S2. Continued

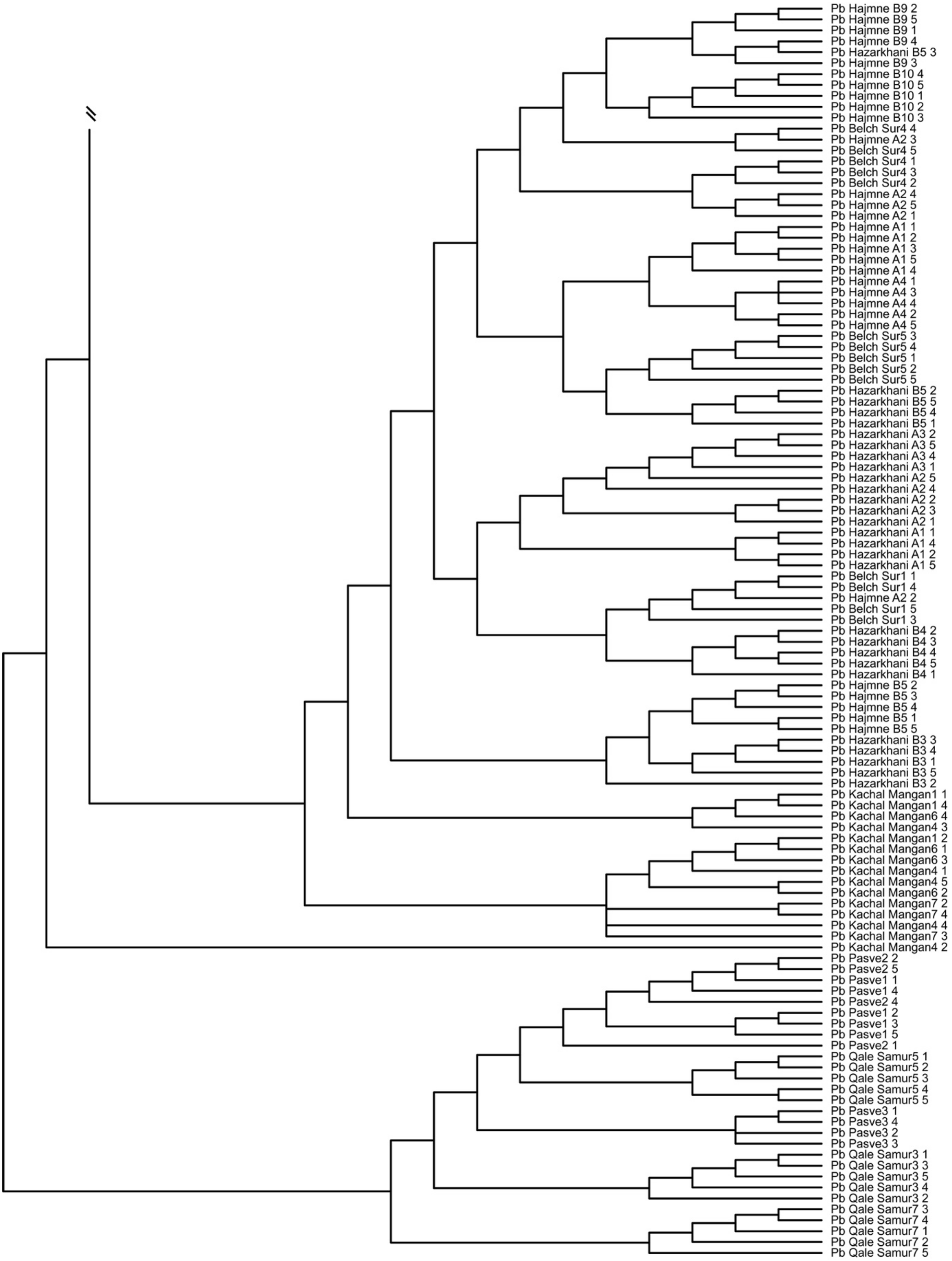

Figure S3. Strict consensus derived from 120 MP trees resulting from the parsimony analysis of the GBS dataset, including 244 *P. bracteatum* individuals.

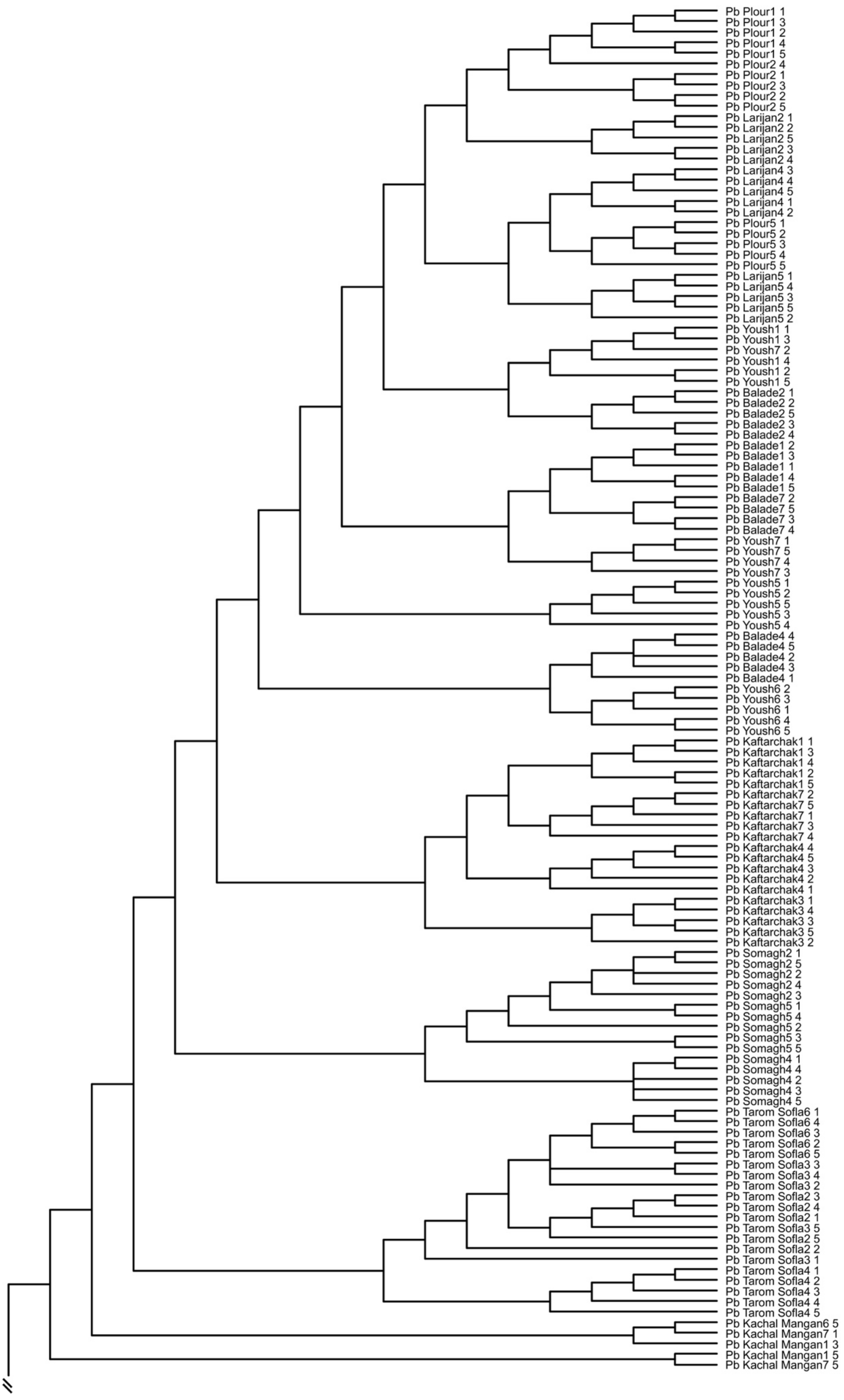

Figure S3. Continued

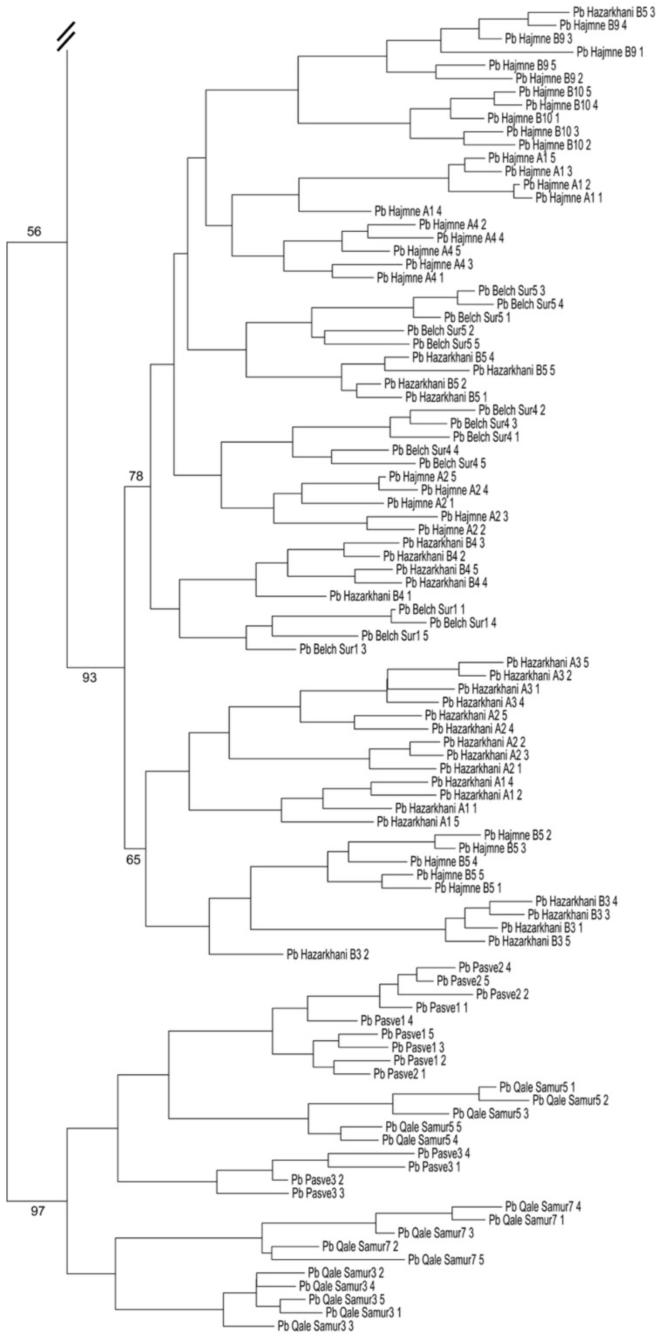

Figure S4. Maximum-likelihood tree for the GBS dataset excluding *P. orientale*. Numbers along branches on the backbone of the tree depict bootstrap support values (≥50%).

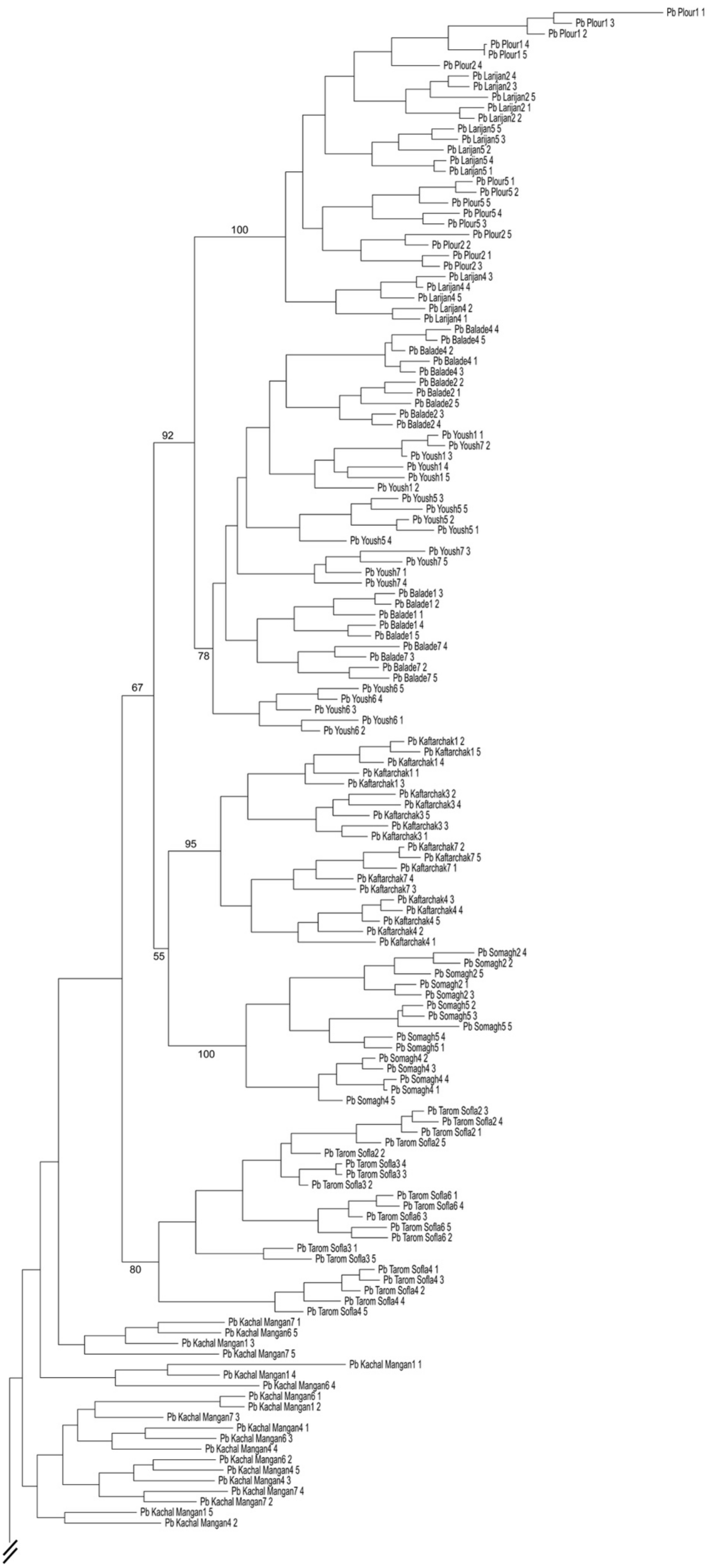

Figure S4. Continued

7b.balade

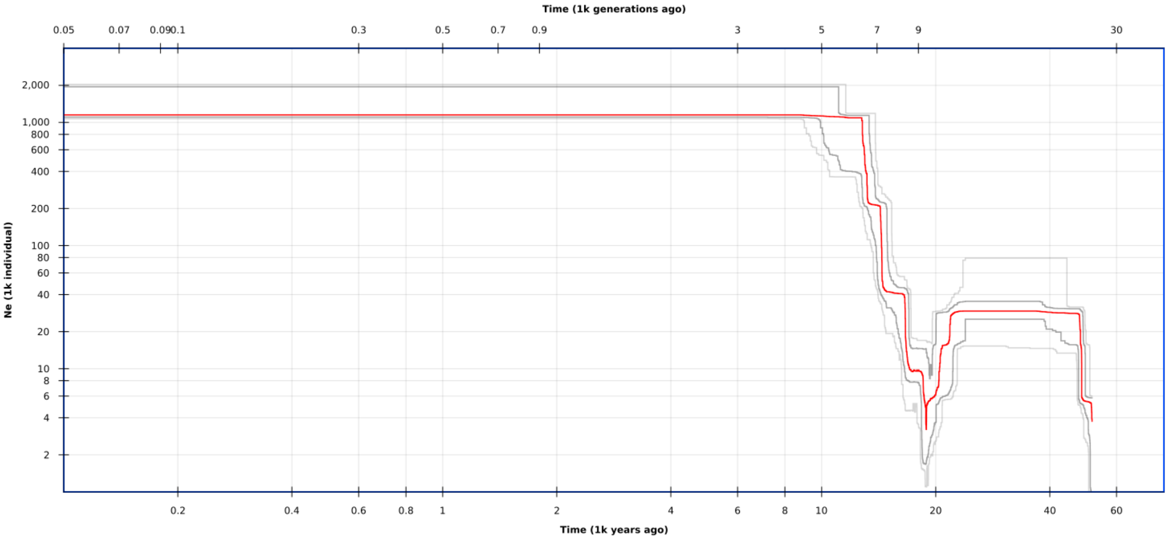

7b.belchsur

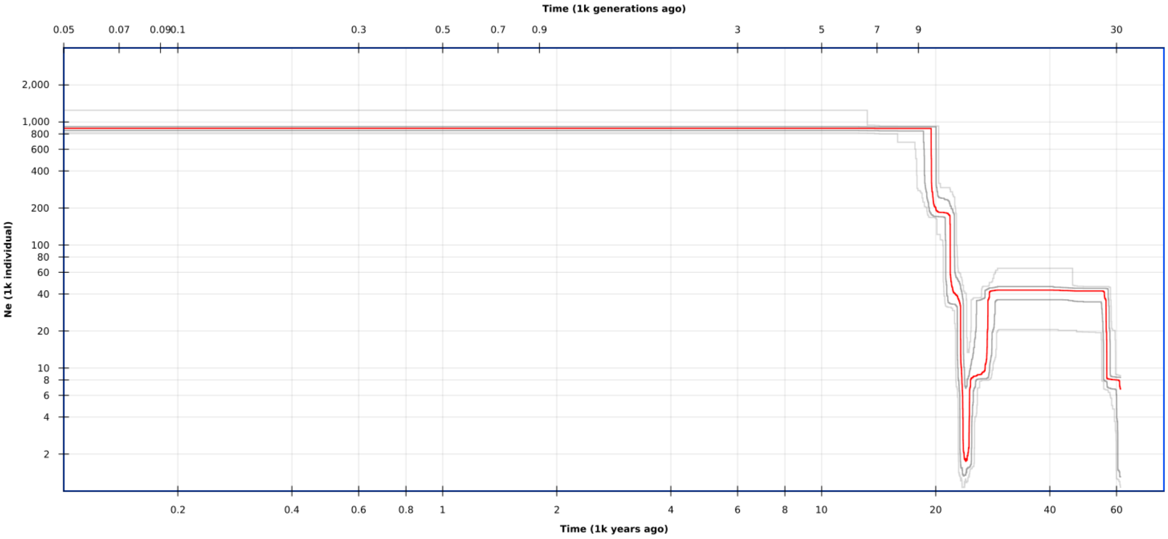

7b.hajmne

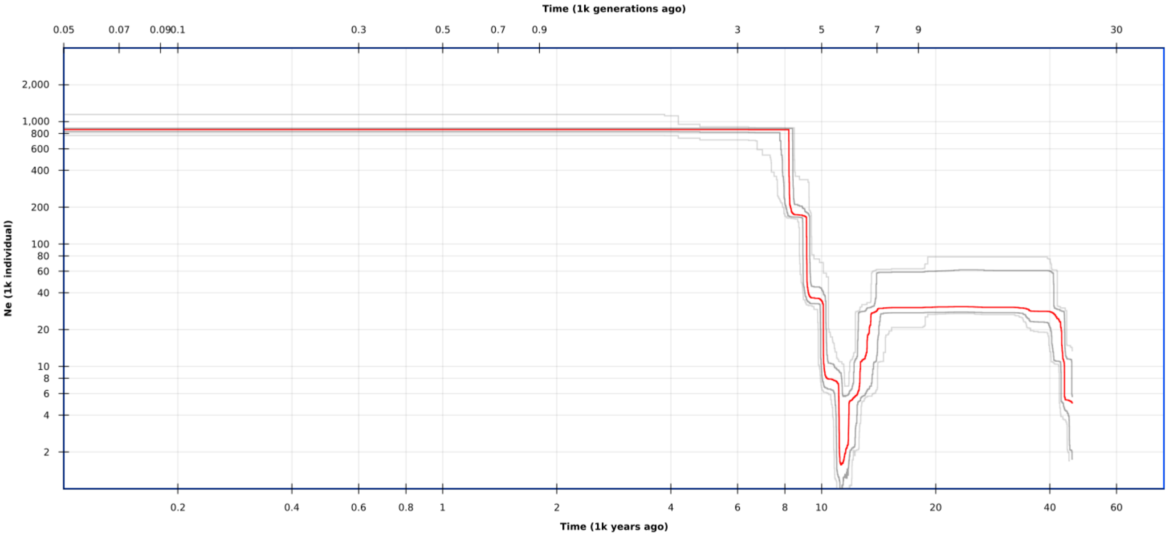

7b.hazarkhani

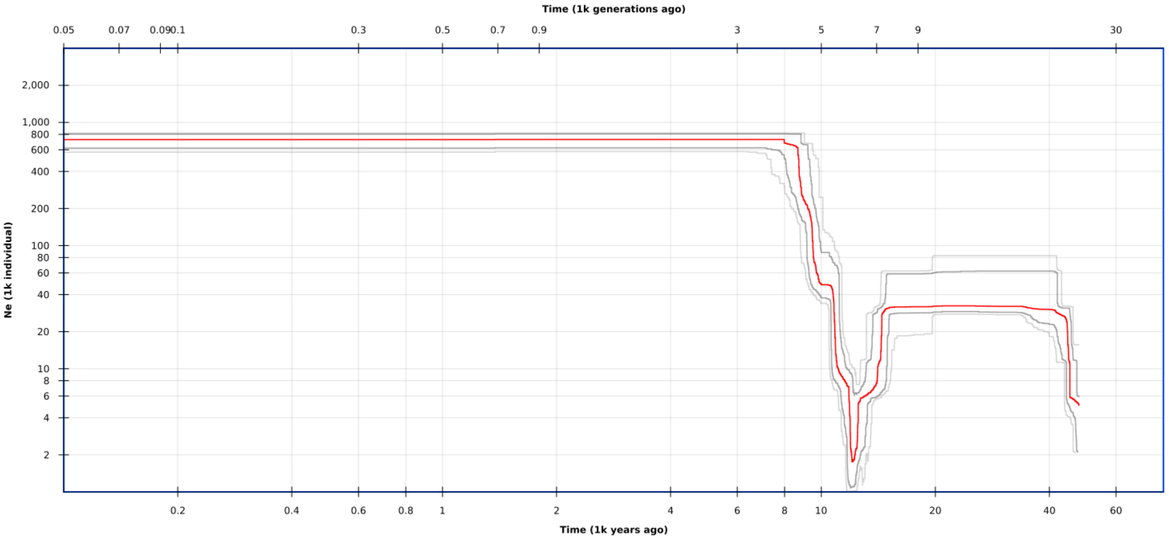

7b.kachalmangan

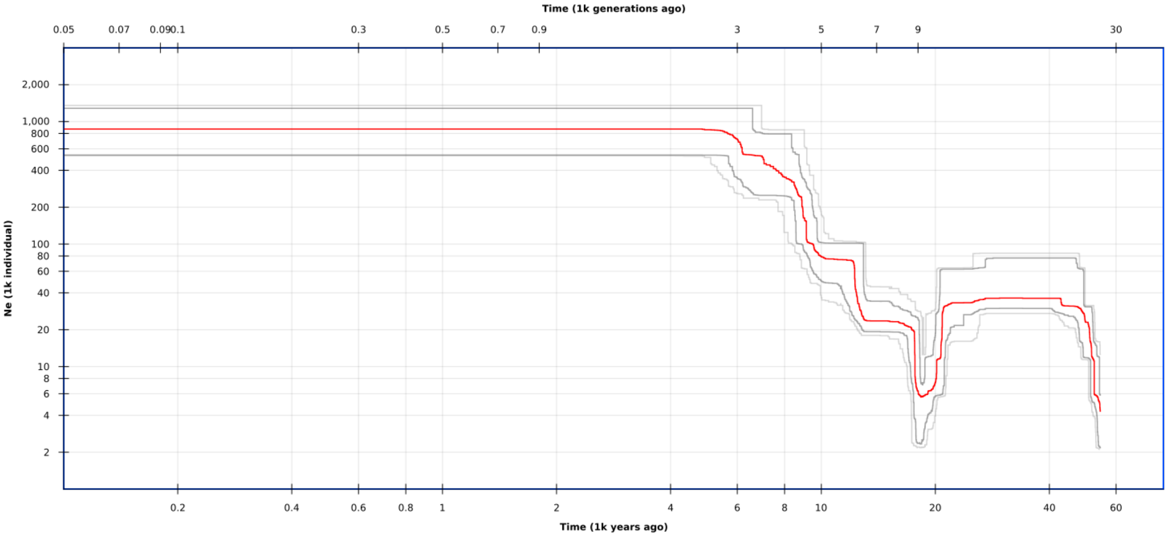

7b.kaftarchak

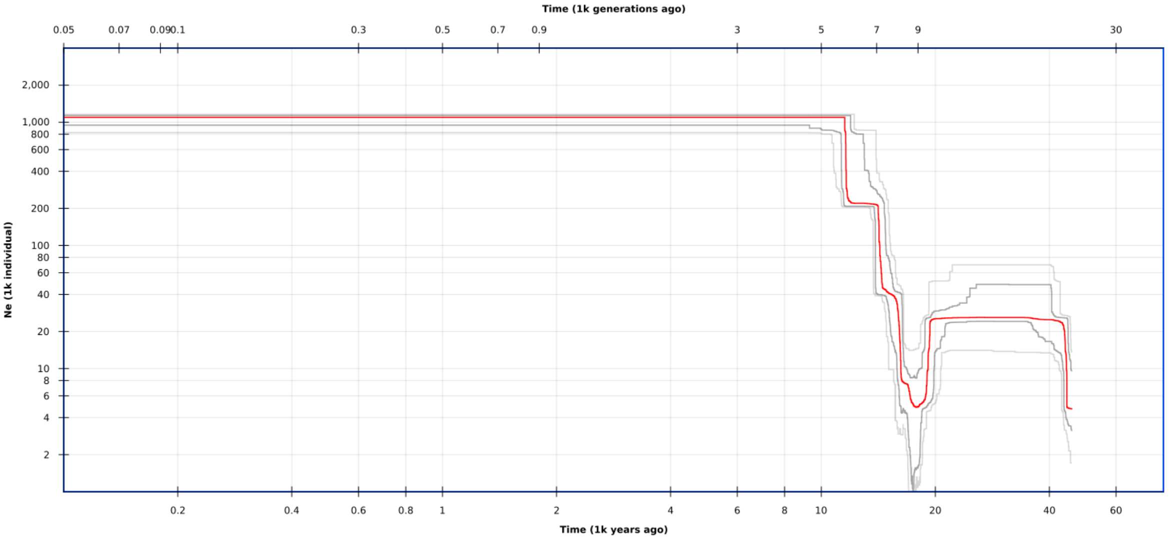

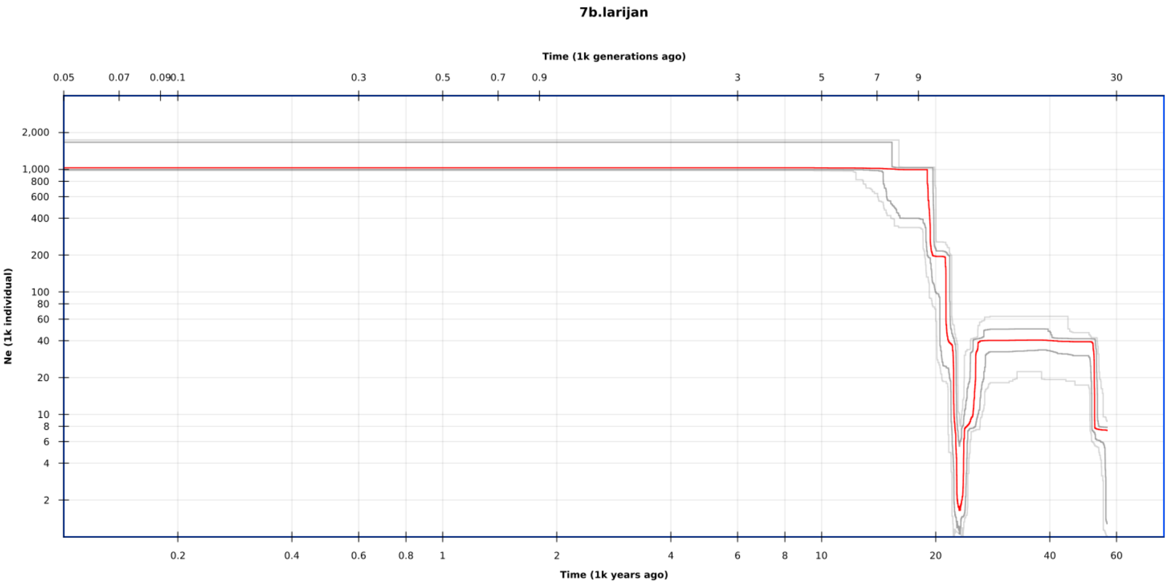
7b.larijan
Time (1k generations ago)
0.05
0.07
0.09
0.1
0.3
0.5
0.7
0.9
3
5
7
9
30
Ne (1k individual)
2,000
1,000
800
600
400
200
100
80
60
40
20
10
8
6
4
2
0.2
0.4
0.6
0.8
1
2
4
6
8
10
20
40
60
Time (1k years ago)

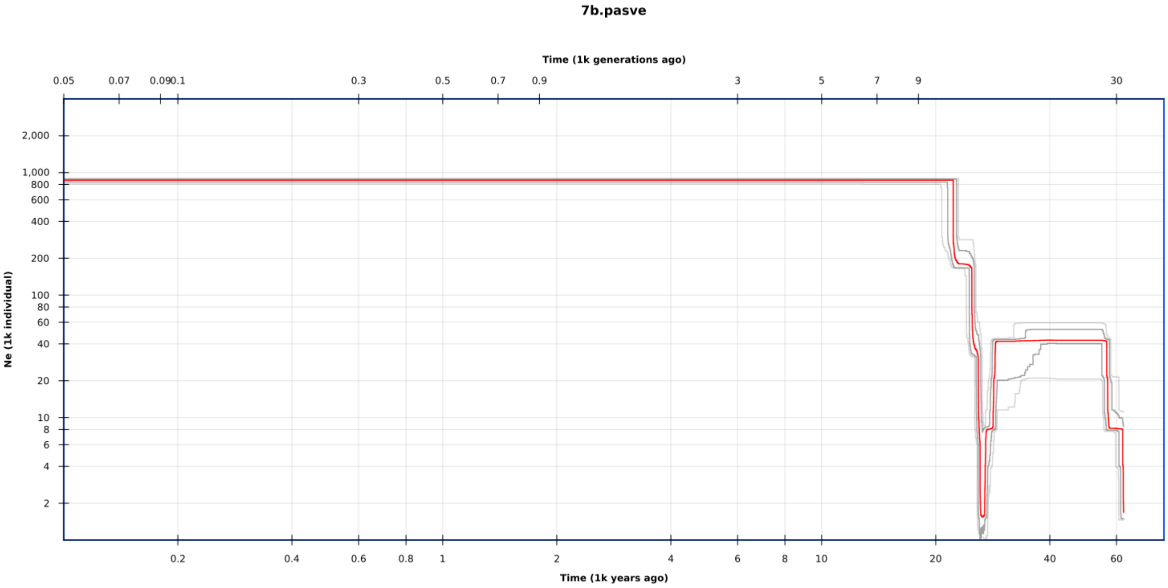
7b.pasve
Time (1k generations ago)
0.05
0.07
0.09
0.1
0.3
0.5
0.7
0.9
3
5
7
9
30
Ne (1k individual)
2,000
1,000
800
600
400
200
100
80
60
40
20
10
8
6
4
2
0.2
0.4
0.6
0.8
1
2
4
6
8
10
20
40
60
Time (1k years ago)

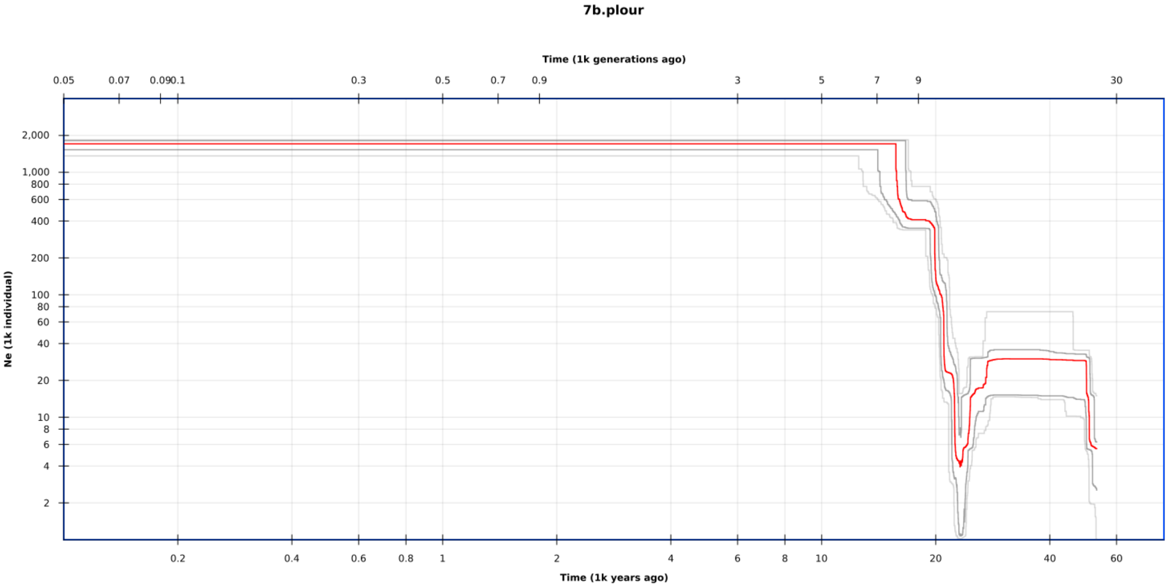
7b.plour
Time (1k generations ago)
0.05
0.07
0.09
0.1
0.3
0.5
0.7
0.9
3
5
7
9
30
Ne (1k individual)
2,000
1,000
800
600
400
200
100
80
60
40
20
10
8
6
4
2
0.2
0.4
0.6
0.8
1
2
4
6
8
10
20
40
60
Time (1k years ago)

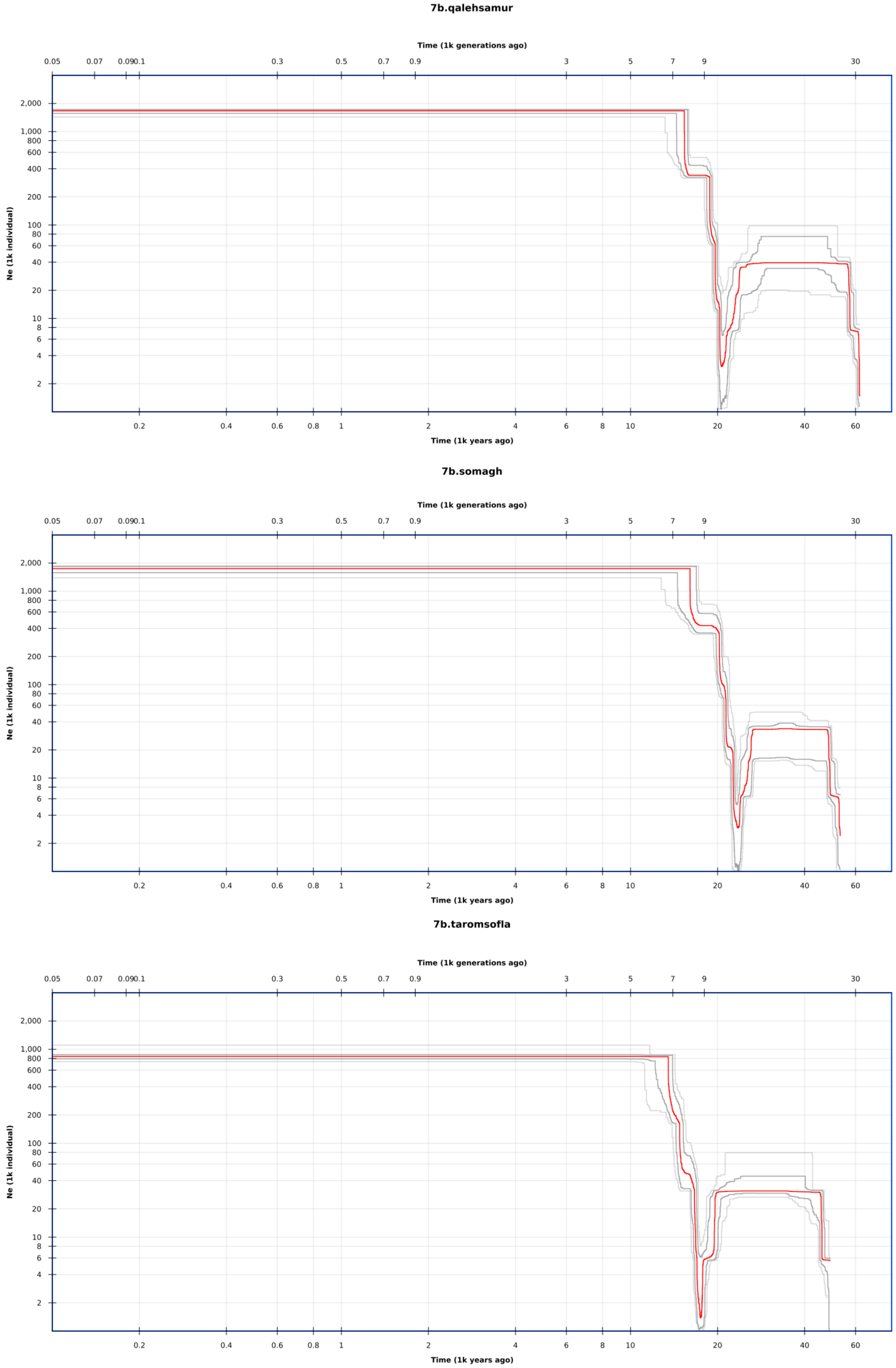

Figure S5. Modelled demographic histories of *Papaver bracteatum* populations, based on GBS data. Population identities are given above the plots. N_e stands for effective population size.

Table S1. Weighted pairwise comparison of genetic differentiation among 13 populations of *P. bracteatum*

	Bal	Bsr	Hjn	Hkn	KMn	Kck	Ljn	Psv	Plr	QSr	Sgh	TSl	Ysh
**Bal**	0												
**BSr**	0.176	0											
**Hjn**	0.161	0.026	0										
**Hkn**	0.155	0.047	0.026	0									
**KMn**	0.139	0.071	0.065	0.061	0								
**Kck**	0.154	0.196	0.175	0.166	0.155	0							
**Ljn**	0.154	0.223	0.200	0.195	0.183	0.200	0						
**Psv**	0.218	0.170	0.156	0.151	0.118	0.251	0.260	0					
**Plr**	0.154	0.218	0.195	0.190	0.183	0.202	0.038	0.260	0				
**QSr**	0.195	0.138	0.132	0.125	0.096	0.223	0.239	0.072	0.236	0			
**Sgh**	0.181	0.198	0.180	0.169	0.153	0.158	0.238	0.253	0.234	0.217	0		
**TSl**	0.168	0.169	0.154	0.149	0.118	0.162	0.218	0.212	0.216	0.188	0.148	0	
**Ysh**	.0.034	0.168	0.154	0.149	0.133	0.147	0.147	0.212	0.148	0.189	0.170	0.161	0

Table S2. Pairwise F_{ST} values between Kachal Mangan and other population groups according to the clustering in the admixture analysis

	Plr/Ljn	Ysh/Bal	Kck	Sgh	TSl	BSr/Hjn/Hkn	Psv/QSr
**KMn**	0.185	0.151	0.155	0.153	0.118	0.056	0.089
